# Protocol for preparing and characterizing samples for combined microsecond freeze-hyperquenching and electron paramagnetic resonance spectroscopy

**DOI:** 10.1016/j.xpro.2026.104405

**Published:** 2026-02-27

**Authors:** Joshua L. Wort, Tobias Hett, Adrian Haardt, Olivia Kendall, Hamed Alaei, Olav Schiemann, U. Benjamin Kaupp

**Affiliations:** 1 Clausius Institute for Physical and Theoretical Chemistry, Rheinische Friedrich-Wilhelms-Universität Bonn, Wegelerstraße 12, 53115 Bonn, Germany

**Keywords:** Biophysics, Protein Biochemistry, Protein expression and purification, Structural Biology

## Abstract

Microsecond freeze-hyperquenching (MHQ) coupled with pulsed electron-electron double resonance spectroscopy (PELDOR) can resolve conformational changes in biomolecules over space and time. Here, we present a protocol for preparing MHQ samples of a cyclic nucleotide-binding domain by rapid mixing with cyclic adenosine monophosphate. Further, we outline the acquisition and analysis of PELDOR data on MHQ samples and study the dynamics of protein-ligand interactions. This protocol can also be extended to other freeze-quench techniques beyond MHQ and other biomolecules.

For complete details on the use and execution of this protocol, please refer to Hett et al.^1^

## Highlights

• Optimization of microsecond freeze-hyperquenching sample preparation
• Spatiotemporal resolution of protein dynamics by double electron-electron resonance
• Extraction of kinetic parameters from freeze-quench pulse dipolar spectroscopy data
• Procedures for microsecond freeze-hyperquench device handling and ease of use

## Abstract (editor highlights)

Publisher’s note: Undertaking any experimental protocol requires adherence to local institutional guidelines for laboratory safety and ethics.

## Graphical abstract

**Figure:**
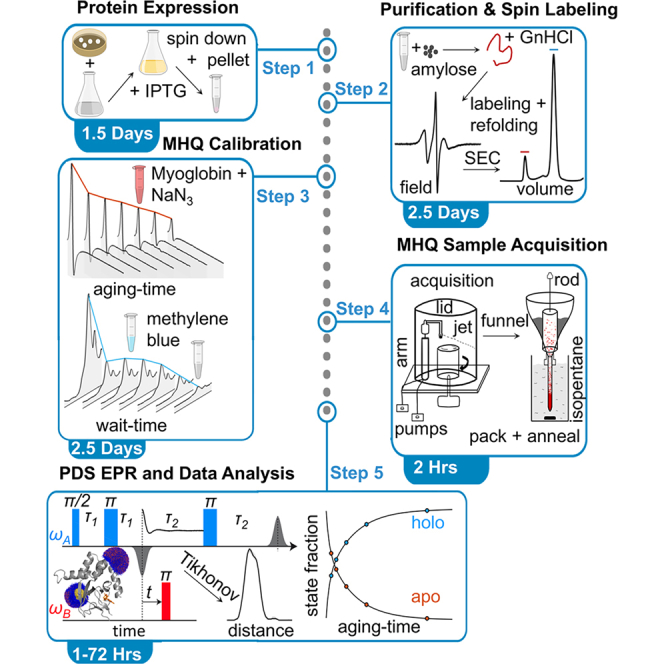

## Before you begin

**Timing: ∼7 days**

This protocol describes the preparation of microsecond freeze-hyperquenching (MHQ) samples, the acquisition of continuous-wave electron paramagnetic resonance (CW-EPR) and pulsed electron-electron double resonance (PELDOR) (a.k.a. double electron-electron resonance (DEER)) measurements on these samples, and the kinetic analysis of the resulting data to study ligand-induced conformational changes in proteins. The approach is robust and well-suited for characterizing protein-ligand interactions that occur on timescales between 80 μs and 20 ms. The analysis relies on the assumption of pseudo-first order kinetics, meaning that the ligand is present in large excess compared to the protein. This requirement is especially critical for reaction times < 100 μs, as the binding equilibrium between ligand and protein must be reached before the sample is cryo-quenched on a cold-plate. As an example, we study the cyclic nucleotide-binding domain (CNBD) of the K^+^ ion channel MloK1 from *Mesorhizobium loti*, interacting with cyclic adenosine monophosphate (cAMP). To monitor conformational changes, exogenous cysteines were introduced at residues E289 and I340 by site-directed mutagenesis and spin-labeled with MTSSL, yielding the R1 side chain. Binding of cAMP causes displacement of an α-helix, resulting in a measurable change in the distance between the spin labels that can be detected by PELDOR. This protein construct was chosen because the interspin distances in the unbound (*apo*) and bound (*holo*) states are relatively short – ∼4.0 nm and ∼2.2 nm, respectively – yielding a substantial distance change (Δ*r*) of ∼1.8 nm.

### Innovation

The function of biomolecules such as proteins and oligonucleotides is closely linked to their conformational dynamics. Thus, for an in-depth understanding of biomolecular function, tracking conformational changes over space-and-time is crucial. When coupled with fast freeze-quench techniques such as microsecond freeze-hyperquenching (MHQ), electron paramagnetic resonance (EPR) experiments like pulsed electron-electron double resonance (PELDOR) can provide spatio-temporal resolution accessing conformational transitions in the Angstrom and microsecond regimes.

While freeze-quench techniques combined with EPR spectroscopy are frequently used to study the dynamics of biomolecules, a comprehensive protocol outlining crucial points of sample preparation and the subsequent acquisition and analysis of EPR data is lacking to date. A detailed, standardized, and structured workflow will create transparency on successful experimental procedures and highlight those that will not work, ensure reproducibility of results, and ultimately make the technique more easily accessible to a broad range of researchers in the field of biophysics, chemistry, and the life sciences.

Initial MHQ reports date back to 2004,^2^ herein we provide a detailed workflow for coupling MHQ with PELDOR, an innovative combination introduced by Hett et al.,^1^ using a protein-ligand model system. We discuss all steps from protein expression, purification, and spin-labeling, through to MHQ sample preparation, PELDOR measurements of MHQ samples, data interpretation and kinetic parameterization. We highlight steps identified as crucial for optimal MHQ sample preparation and subsequent MHQ handling. Indeed, MHQ/PELDOR has broader applicability in near-native studies of biomolecules by EPR spectroscopy in absence of cryoprotectant, as previously demonstrated for sub-millisecond freeze-quench EPR spectroscopy.^3^

### Institutional permissions (if applicable)

Not applicable.

### Expression, purification, and spin-labeling of the *M. loti* MloK1 CNBD

**Timing: ∼4 days**

The following section outlines the transformation of the pMalc2X plasmid vector and the recombinant expression of the MloK1 CNBD construct (C263S/E289C/C331S/I340C) in BL21(DE3) pLysE *E. coli* cells. To improve solubility and the protein yield, the CNBD is fused to an N-terminal maltose-binding protein (MBP) tag. Subsequently, the section details protein purification performed by a combination of affinity- and size-exclusion chromatography, and spin-labeling with the nitroxide MTSSL facilitated through partial protein denaturation.

1. Preparation steps prior to CNBD expression.
  a. Prepare 10 mL of ampicillin stock solution (100 mg mL^−1^) using filtered MilliQ H_2_O.
  b. Prepare 10 mL of isopropyl β-D-1-thiogalactopyranoside (IPTG) stock solution (1 M) using filtered MilliQ H_2_O.
  c. Prepare Luria-Bertani (LB) agar plates supplemented with ampicillin (100 μg mL^−1^).
  d. Prepare 50 mL of LB medium in a Duran flask, autoclave at 121°C for 15 min, and store at 4°C.
    ***Note:*** To prepare Luria-Bertani (LB) medium^4^ dissolve: 10 g tryptone, 5 g yeast extract, and 10 g NaCl in 1 L MilliQ H_2_O, add 200 μL 5 M NaOH to adjust pH to 7.0. For LB agar plates, add 12 g of agar per 1 L of medium.
  e. Prepare 5 × 1 L batches of Terrific Broth (TB) medium in 2 L growth flasks, cover with aluminium foil, and sterilize by autoclaving.
  f. Use site-directed mutagenesis^5^ to introduce the specific mutations required for thiol-based spin labeling. This includes removing exposed native cysteine residues and inserting exogenous cysteine residues at designated positions.
    ***Note:****In silico* spin-labeling toolboxes such as mtsslWizard,^6^^,^^7^ MMM,^8^^,^^9^ and chiLife^10^ can assist in designing protein constructs suitable for monitoring conformational changes by PELDOR. The CNBD construct used in this protocol (E289R1/I340R1) was selected because the *apo*- and *holo*-state distance distributions are well-separated (Δr > 0.6 nm), as identified using difference-distance maps within mtsslWizard.
    **CRITICAL:** Verify the fidelity of the plasmid DNA sequence by Sanger sequencing and assess plasmid DNA quality using a 1% (w/v) agarose gel (can load 20 ng of DNA). Finally, extract plasmid DNA using a Miniprep kit before proceeding to step 2 of this preparation section.
    **CRITICAL:** The steps 2c-4 should be performed using sterile (i.e., aseptic) technique to minimize microbial contamination risk.^11^ Before proceeding, disinfect surfaces to be used with 70% (v/v) EtOH, procure appropriate personal protective equipment (PPE) (i.e., nitrile gloves and lab coat), work near an open flame (i.e., via a Bunsen burner), and reduce exposure-time to open air.
2. Transformation of *E. coli* cells with plasmid DNA.
  a. Thaw a 50 μL aliquot of BL21(DE3) pLysE *E. coli* cells on ice, then add approx. 50 ng of purified plasmid DNA.
  b. Incubate the sample on ice for 30 min, then transfer the cells to a 42°C water bath for 45 s to heat-shock, then return them to ice for 2 min.
  c. Add 200 μL of LB medium at ambient temperature.
  d. Incubate at 37°C with constant agitation (800 rpm) for 1 h in a shaking incubator.
  e. Plate the suspension onto the LB agar containing ampicillin (100 μg mL^−1^) and incubate for 16 h at 37°C.
3. Preparation of a starter culture.
  a. Select a single colony of transformed *E. coli* cells and inoculate 50 mL of LB medium containing ampicillin (100 μg mL^−1^) in a 500 mL baffled flask.
  b. Incubate for 16 h at 37°C with constant agitation (180 rpm) in a shaking incubator.
4. Large-scale preparation. Inoculate 5 × 1 L flasks of TB medium, each containing ampicillin (100 μg mL^−1^) with the starter culture. Ensure the optical density (OD_600_) is approx. 0.2 at the time of inoculation.

***Optional:*** For enhanced control of aeration and agitation, a fermenter may be used instead of a shaking incubator.

5. Grow the cells in a fermenter at 37°C with agitation (1,500 rpm) and a constant airflow (7 mL min^−1^) until OD_600_ reaches 2.5–3.0.
6. Induction of protein expression. Reduce the temperature to 20°C and add IPTG to a final concentration of 0.8 mM.
7. Post-induction incubation. Continue incubation at 20°C, 1,500 rpm, 7 mL min^−1^ airflow for an additional 20 h.
8. Cell harvesting and storage. Harvest the cells by centrifugation (6000 × g, 10 min, 4°C), discard the supernatant, and store the cell pellet at −20°C until further use.
9. Prepare the following solutions required for purification and spin-labeling of the CNBD. Store them at 4°C, unless otherwise specified.
  ***Note:*** All buffer solutions in step 9 are protonated, unless explicitly stated otherwise.
  a. 1 × Phosphate-Buffered Saline (PBS): 1 L containing 150 mM NaCl, 2.7 mM KCl, 10 mM Na_2_HPO_4_, 1.8 mM KH_2_PO_4_, at pH 7.4.
  b. 1 mL aliquots of 1 M dithiothreitol (DTT); store at −80°C.
  c. 250 mL of 1 × PBS, supplemented with 10 mM DTT and EDTA-free protease inhibitor cocktail; these additives are added immediately before use.
  d. Refolding buffer: 500 mL containing 7 mM Na_2_HPO_4_, 3 mM NaH_2_PO_4_, 150 mM NaCl, 500 mM L-arginine, 10 mM EDTA, at pH 7.4.
  e. Denaturing buffer: 500 mL containing 7 mM Na_2_HPO_4_, 3 mM NaH_2_PO_4_, 150 mM NaCl, 6 M guanidine, at pH 7.4.
  f. MTSSL spin label: Prepare 25 μL aliquots of 100 mM S-(1-oxyl-2,2,5,5-tetramethylpyrroline-3-methyl)methanethiosulfonate, dissolved in DMSO (26.44 mg in 1 mL) and store at −80°C.
  g. 70% (v/v) EtOH: 50 mL for cleaning the sonicator.
10. Amylose resin preparation: decant the resin into a 10 mL volume gravity column, recharge, wash, and equilibrate the amylose resin (0.2 mL g^−1^ of cell pellet) with:
  a. Three column volumes (3 × 10 mL) of MilliQ H_2_O to remove the storage solution (20% (v/v) EtOH).
    ***Note:***Step 10b is only required when recharging the column and should not be performed more than approx. five times.
  b. Wash resin with three column volumes (3 × 10 mL) of 0.1% (v/v) sodium dodecyl sulfate (SDS) solution, followed by five column volumes (5 × 10 mL) of 1 × PBS (see step 9a).
  c. Store the resin at 4°C.
11. Freeze-dry 20 mL of refolding buffer (see step 9d), seal it under Parafilm, and store at ambient temperature (18°C–25°C).

***Note:*** The following section outlines the purification and spin-labeling of the MloK1 CNBD, optimized for clarity and ease of use. The procedure combines protein purification by affinity chromatography, protein denaturation and refolding, and size-exclusion chromatography. Spin labeling is performed using the thiol-specific nitroxide MTSSL to introduce the paramagnetic R1 side chain. While demonstrated for the E289C/I340C construct, this method is also suitable for other double-cysteine CNBD mutants.

12. Cell pellet thawing, resuspension, and lysis.
  a. Thaw the cell pellets from step 8 and resuspend in 1 × PBS buffer (see step 9a) at a 3:1 volume-to-mass ratio.
  b. Add DTT (final concentration, 10 mM) and a protease inhibitor cocktail (one tablet per 50 mL) to the 1 × PBS buffer (see step 9c) before use.
  c. Lyse the cells by sonication (4 min, 40% of max. amplitude), ensuring that the sonicator probe is cleaned with 70% (v/v) EtOH (see step 9g) before and after use.

***Note:*** Perform sonication on ice to prevent overheating.

13. Centrifuge the lysate at 40,000 × g, for 60 min at 4°C. Collect the supernatant for further purification.
14. Amylose affinity chromatography and protein denaturation.
  a. Decant equilibrated amylose resin (see step 10) (0.2 mL g^−1^ of cell pellet) into the supernatant and incubate for 30 min at 4°C with constant agitation.
  b. Sediment the amylose resin by centrifugation at 4000 × g for 10 min at 4°C, discarding the supernatant.
  c. Resuspend the amylose resin in supplemented 1 × PBS buffer (see step 9c) (2.5 mL per mL of resin) and repeat step 14b to remove DTT and non-specific *E. coli* proteins. Repeat this procedure of resuspending and centrifuging the resin five times.
  d. Exchange the amylose resin into denaturing buffer (see step 9e) (2.5 mL per mL of resin), centrifuge (4000 × g, 10 min, 4°C), and collect the supernatant.
    ***Note:*** The denaturing buffer unfolds the N-terminal MBP tag, preventing its association with the amylose resin. This step also removes endogenous cAMP from *E. coli* cells bound to the CNBD during purification and can improve spin-labeling efficiency, particularly if the labeling site is buried within the protein.
  e. Add MTSSL at a 5-fold molar excess relative to the protein and incubate for 12–16 h at 4°C.
    **CRITICAL:** Protect the spin-labeling mixture from light by wrapping a 15 mL Falcon tube in aluminium foil, as MTSSL is light-sensitive.
15. Wash out unbound label.
  a. Separate free cAMP and excess MTSSL from the unfolded protein using a spin column (15 mL volume, 30 kDa molecular weight cutoff) (4000 × g, 5 min, 4°C).
  b. Repeat the washing step with 4 mL of denaturing buffer (see step 9e) at least three times concentrating the sample to approx. 1 mL.

***Note:*** To confirm that cAMP has been efficiently removed from the CNBD, measure the absorbance ratio at 260 nm and 280 nm. A ratio close to 0.8 indicates a cAMP-free CNBD.

**Figure 1:**
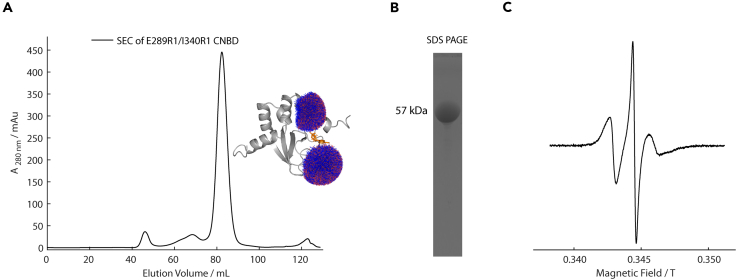
Purification and CW-EPR of CNBD construct C263S/E289R1/C331S/I340R1 (A) Size-exclusion chromatogram of the CNBD construct C263S/E289R1/C331S/I340R1, indicating a mono-disperse peak at approx. 84 mL elution volume. The *holo*-state (PDB: 2K0G) with R1 rotamer clouds generated by mtsslWizard is shown as an inset figure. (B) SDS-PAGE gel image of CNBD construct E289C/I340C after the refolding step. The band corresponds to a molecular weight of approx. 57 kDa (i.e., the sum of the E289R1/I340R1 CNBD construct and the maltose-binding protein fusion tag). Note that the gel image was cropped from the left; four lanes including samples of marker, supernatant, amylose affinity purification, and protein refolding were removed for conciseness. (C) X-band CW-EPR spectrum of the E289R1/I340R1 CNBD construct, indicating immobilization of the spin label and quantitative labeling.

16. Protein refolding procedure:
  a. Add 3 mL of the CNBD solution dropwise using a Pasteur pipette to 40 mL of ice-cold refolding buffer (see step 9d), mixing gently by stirring.
  b. Centrifuge the mixture at 4000 × g for 5 min at 4°C using a spin column with a 30 kDa molecular weight cutoff.
  c. Repeat step 16b as needed to reduce the total volume to 2–4 mL.
17. Size-exclusion chromatography:
  a. Attach the pre-equilibrated HiLoad 16/60 Superdex 200 pg column to the ÄKTA system and equilibrate with two column volumes of 1 × PBS buffer (see step 9a). This process takes several hours (3–4 h).
  b. Rinse the sample loop of the ÄKTA system with two loop volumes of degassed MilliQ H_2_O, followed by two loop volumes of degassed 1 × PBS buffer (see step 9a).
    ***Note:*** Buffer degassing was performed via vacuum pumping into a Duran flask with O-ring to ensure vacuum sealing. During degassing buffers were agitated via stirring with a magnetic stir bar.
    **CRITICAL:** Avoid introducing air into the column at all stages, including when connecting the column to the ÄKTA system and loading the CNBD solution onto the column through the sample loop.
  c. Using a cannula, draw the CNBD solution into a 5 mL syringe. Discard the cannula after use.
  d. Connect the syringe to the sample loop of the ÄKTA system and load the loop with the CNBD solution, ensuring the loop volume is at least twice the sample volume. Take care to avoid air bubbles while loading.
  e. Load the CNBD solution onto the column and elute with refolding buffer (see step 9d).
  f. Identify CNBD-containing fractions by their monodisperse peak, typically eluting in the size-exclusion chromatogram at approx. 84 mL (see section expected outcomes, Figure 1).
  g. Pool these fractions and concentrate them using a fresh spin column (30 kDa molecular weight cutoff) at 4000 × g, 4°C. Adjust the final protein concentration to 300 μM, freeze 200 μL aliquots in N_2(l)_, and store at −80°C.
18. Assessment of labeling efficiency:
  a. Use a Nanodrop spectrophotometer to measure A_280nm_ and calculate the CNBD concentration (using ε_280nm_ = 71,850 M^−1^ cm^−1^, calculated from the Expasy ProtParam tool^12^). Dilute an aliquot to approx. 100 μM for CW-EPR measurement.
  b. Pipette 15 μL of the ∼100 μM CNBD solution into the lid of a 1.5 mL Eppendorf tube. Fill a 0.6 mm i.d. EPR microcapillary with 10 μL of the solution.
  c. Seal the microcapillary with silicone paste and place it into a 4 mm o.d. X-band EPR tube. Insert the EPR tube into the resonator of the EMXnano CW-EPR spectrometer. For more details, consult the instrument manual.
  d. Tune the spectrometer using the autotuning routine:
    i. Open the tuning panel in the Xenon software controlling the spectrometer. Switch from standby to tune mode in the tuning panel.
    ii. Click “Lock Search” in the tuning panel and wait for the spectrometer to return to “Operate” mode.
    iii. Once the spectrometer returns to “Operate” mode (indicated by a green status indicator in the tuning panel), verify in the main window that the “Matching” counter needle reads 0 μA and the “Matching Fine” counter needle is close to 0%.
      ***Note:*** If either “Matching” or “Matching Fine” value is incorrect, repeat the tuning procedure.
    iv. While in Operate mode, check that the “Operate”, “Levelled”, and “Calibrated” indicators in the main window are all green.
  e. Record a CW-EPR spectrum (the first derivative of the absorbance EPR spectrum) at 298 K using the parameters listed in Table 1.
    ***Note:*** For detailed instructions on setting up the CW-EPR experiment, consult the user manual of the spectrometer.
  f. Determination of the spin concentration:
    i. Access the “Quantitative EPR” tab and open the “SpinCount” function from the left-hand sidebar.
    ii. Click “Define Region” and select the entire spectrum. Click the “Define Region” button again to confirm.
    iii. Adjust the red qualifier bars to mark the baseline region on both sides of the spectrum.
    iv. Click “Double Integration”. The double integral trace of the spectrum will be displayed as a blue line.
      ***Note:*** Record spectra with a 10–20 G baseline on either side of the nitroxide signal to allow for robust baseline correction. Verify that the double integral increases continuously throughout the whole spectrum for reliable results.
    v. Calculate the spin concentration. Click “Calculate”. Enter the following parameters in the dialogue window: “Diameter” (0.6 mm, inner diameter of the microcapillary), “Center” (125 mm, resonator-specific value, 125 mm for EMXnano), and “Length” (approx. 25 mm).
    vi. Confirm by clicking “OK” and record the resulting spin concentration.
  g. Determine the spin-labeling efficiency using Equation 1:
    (Equation 1) $$labeling\;efficiency\left(\%\right)=\frac{c\left(spins\left(\mu M\right)\right)}{n\times c\left(protein\left(\mu M\right)\right)}\times 100$$
    ***Note:****n* is the number of accessible cysteine residues per protein monomer (*n* = 2 for CNBD constructs discussed in this work). Labeling efficiency may vary by construct; but values ≥ 75% are recommended for optimal signal intensity in subsequent PELDOR experiments.
19. Re-dissolve the freeze-dried refolding buffer (see step 9d) in D_2_O.
  a. Centrifuge the CNBD sample in a spin column (30 kDa molecular weight cutoff) at 4000 × g for 5 min at 4°C. Repeat until the final volume is approx. 100 μL.
  b. Add 400 μL of deuterated refolding buffer (see step 9d) and repeat the buffer exchange at least three times.
20. Adjust the CNBD concentration to approx. 300 μM and snap-freeze 200 μL aliquots in N_2(l)_. Store at −80°C.

**Table 1:** Parameters for the CW-EPR measurement at 298 K to determine the spin concentration of the C263S/E298R1/C331S/I340R1 CNBD construct

Parameter	Value
# of scans	10
# of points	1300
Center Field	3445 G (corresponding to g∼2.005)
Attenuation	16 dB (≙ 2.5 mW)
Sweep Width	130 G
Receiver Gain	40 dB
Conversion Time	20.94 ms
Time-constant	20.48 ms
Modulation Amplitude	1 G

### Equilibration, wait-time optimization, and aging time calibration of the MHQ

**Timing: 2.5 days**

The following steps describe how to equilibrate the MHQ into appropriate buffers for operation and storage, optimize the mixer-arm wait-time using methylene blue as a dilution reporter throughout the sample acquisition, and how to calibrate the MHQ aging times using the reaction between metmyoglobin and sodium azide as a “molecular timer”. These experiments verify the reliable functionality of the MHQ and are not required for routine operation.

21. Prepare and filter 1 L each of MilliQ H_2_O, 1× PBS buffer (see step 9a), and 20% (v/v) EtOH into freshly cleaned Duran bottles using 0.45 μm syringe filters and a vacuum pump. Degas the solutions for 5–10 min.

**CRITICAL:** All solutions passed through the MHQ must be filtered (≤0.45 μm pore size) or centrifuged (13,000 × *g*, 5 min, 4°C) to remove particulate matter that could clog the MHQ orifice (20 μm diameter) or HPLC pumps. Troubleshooting 1.

***Optional:*** To ensure the removal of any particulate matter from the interior of the glass bottle, agitate the filtered solution and filter it a second time into the same bottle. This step is recommended if the Duran bottle has been left open prior to use.

***Note:*** After each session at the MHQ, HPLC pumps should be stored in 20% (v/v) EtOH.

22. Equilibrate the MHQ in 1 × PBS buffer (see step 9a).
  a. Check the flow-rate and pressure of the HPLC pumps. Switch on both HPLC pumps (for protein and ligand channel) and set each to a flow rate of 1 mL min^−1^.
    ***Note:*** In 20% (v/v) EtOH, the pressure reading at both pumps should remain below ∼1800 psi. If a single, unbroken stream (jet) is observed from the orifice and if there are no pressure spikes above 2000 psi, stop the flow. If the jet is broken or there are pressure spikes, see troubleshooting 1, troubleshooting 5.
  b. Remove both HPLC tubes from the storage solution (EtOH, 20% (v/v)) and place them into filtered MilliQ H_2_O.
  c. Reset the flow rate to 1 mL min^−1^ for each pump, ensuring that the sample injection valves for both channels are set to “load”. Perform this equilibration for 15 min.
    ***Note:***Steps 23–27 (below) can be performed during step 22c of the equilibration period.
  d. Flush the 1-mL sample loops with 5 mL MilliQ H_2_O to remove any air. Keep the syringes in the sample injection valves to prevent air from re-entering the loops.
  e. After equilibration, set both sample injection valves from “load” to “inject” and verify that the pressure remains stable for at least 5 min.
    ***Note:*** A pressure increase indicates an air bubble or debris in the tubing, a decrease suggests a leak.
  f. When the pressure is stable, return both sample injection valves to the “load” position and stop the flow. Wait for the pressure to drop to 0 psi.
  g. Repeat steps 22c–22e using filtered 1× PBS buffer (see step 9a).
    ***Note:*** As outlined,^1^ MHQ samples are prepared by spraying a mixture of protein and ligand through a nozzle onto the surface of a cryogenically cooled hollow metal cylinder (“cold-plate”), rotating at 7000 rpm, where the mixture freezes within microseconds (see ESI Figure S1). The aging time (*t*_*a*_) of the reactants (protein and ligand) is determined by the distance between the mixer orifice (see ESI Figure S2) and the cold-plate wall. By preparing samples at various distances (various aging times), one can reconstruct protein dynamics from “snapshots” at different time points. Efficient coverage of the cold-plate surface is achieved by moving the micromixer vertically. Synchronizing sample ejection with the vertical position of the mixer is essential to minimize sample loss. A delay (“mixer-arm wait-time”) between sample injection into the HPLC loops and the start of the vertical movement of the mixer is required to account for the transit time of reactants from the injectors to the micromixer. Incorrect mixer-arm wait-time settings may result in spraying the reactants outside the cold-plate and the sample collected on the cold-plate will be diluted by further spraying of buffer.
    **CRITICAL:** Methylene blue is toxic and can be absorbed through the skin. Always wear appropriate PPE, including nitrile gloves and a face shield, particularly during syringe filtration steps.
23. Prepare a 4 mM methylene blue (MB) dye stock:
  a. Vacuum-filter 100 mL of MilliQ H_2_O using a 0.45 μm filter.
  b. Accurately weigh 128 mg of methylene blue (molar mass = 319.85 g mol^−1^) and dissolve it in the filtered MilliQ H_2_O using a magnetic stirring rod and plate.
  c. Filter the methylene blue stock solution using a 0.45 μm syringe filter, collecting the filtrate in two 50 mL Falcon tubes. Aliquot 1 mL into an Eppendorf tube.
  d. Determine the absorbance of the solution at 280 nm using a Nanodrop spectrophotometer (extinction coefficient of methylene blue *ε*_280*nm*_ = 28,000 M^−1^ cm^−1^). Confirm that the concentration is approx. 4 mM.
    ***Note:*** The absorbance peak at 280 nm corresponds to monomeric methylene blue, while the absorbance peak at *λ*_*max*_ indicates oligomeric (dimeric and tetrameric) forms.^13^ Although the exact concentration is not critical, a 4 mM solution is sufficient for UV-visible detection, even after a 20-fold dilution.
  e. Store the methylene blue stock solution at 4°C in Falcon tubes sealed with Parafilm.
24. Switch on the MHQ device, including the cold-plate motor, the mixer arm, and the vacuum pump. Open the gas ballast valve of the vacuum pump and allow the system to warm up for 20 min. Equilibrate the MHQ in MilliQ H_2_O (see steps 22c and 22d).

**Figure 2:**
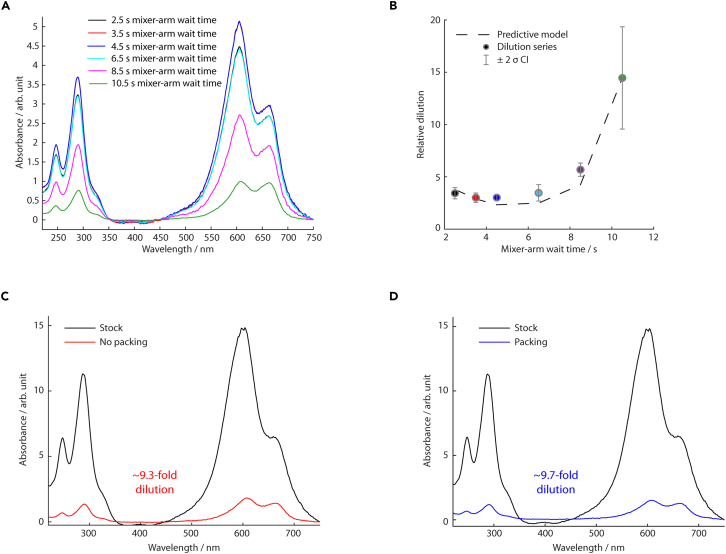
MHQ mixer-arm wait-time optimization (A) UV-visible spectra (mean of *n*=3 replicates) of MHQ-shot methylene blue as a function of the mixer-arm wait-time. (B) The dilution profile (i.e., the quotient of the measured A_280nm_ of the methylene blue solution collected from the MHQ and the methylene blue stock solution) as a function of the mixer-arm wait-time (scatter plot). Error bars indicate the 2σ confidence intervals. The black dotted line is the univariate fit of Equation 4 to the experimental data. The color scheme is consistent with panel (A). (C) UV-visible spectra (mean of *n*=3 replicates) of methylene blue after cold-shooting without packing (mixer-arm wait-time = 4.5 s). (D) UV-visible spectra (mean of *n*=2 replicates) of methylene blue after cold-shooting with packing (mixer-arm wait-time = 4.5 s).

***Note:*** For MHQ wait-time optimization, perform “warm-shooting” (i.e., do not pre-cool the cold-plate with N_2(l)_) and do not pack the sample into an EPR tube. This approach excludes dilution from condensation during sample deposition onto the cold-plate and tube packing (see expected outcomes section, Figure 2).

25. Load 100 μL of methylene blue solution into the ligand syringe and 100 μL of MilliQ H_2_O into the protein syringe. Insert the syringes into their respective injection valves and ensure both valves are set to the “load” position.

***Note:*** The sample-loop volume should closely match the dye volume to minimize the dead volume and reduce dilution caused by lamellar flow during injection of the dye into the sample loops.

26. Launch the software for controlling the step motor that moves the mixer-arm (herein, LinControl), and the software controlling the cold-plate motor (herein, IndraWorks).
  ***Alternatives:*** Any other program capable of controlling the mixer arm can be used as an alternative to the LinControl software.
  a. Swing the mixer-arm over the cold-plate. In LinControl (or equivalent software), set the values given in Table 2 for “home position”, “top velocity”, “acceleration”, and “move absolute”.
  b. Verify that the mixer-arm descends to the center of the cold-plate and holds its position.
  c. To determine the appropriate aging time, use an electronic caliper to measure the distance from the nozzle outlet to the wall of the cold-plate.
  d. Adjust this distance according to Table 3, based on the desired aging time. The aging time is calculated using Equation 2^1^^,^^2^
    (Equation 2) $$t_{a}=t_{m}+0.9t_{t}+t_{c}$$
    ***Note:*** Here *t*_*m*_ is the mixing time (i.e., the residence time of the sample in the micromixer), *t*_*t*_ is the transport time from the nozzle outlet to the cold-plate, and *t*_*c*_ is the cryofixation time (i.e., the freezing time on the cold-plate). Typically, *t*_*m*_ is negligible (<1 μs), and *t*_*c*_ is 40 μs.^1^^,^^2^ The prefactor of 0.9 for the transport time was derived from laser Doppler anemometry measurements.^2^
  e. The transport time *t*_*t*_ is determined by the jet length *l*_*jet*_ and the total flow rate *f*, as shown in Equation 3
    (Equation 3) $$t_{t}=\frac{l_{jet}}{f}$$
    This value can be adjusted by varying the jet length.
    ***Note:*** These aging times are calculated for a total flow rate (taking both the protein channel and the ligand channel into account) of 2 mL min^−1^ and an orifice diameter of 20 μm. Distances shorter than 3 mm may cause copper abrasion from the nozzle, resulting in sample contamination.
  f. Return the mixer-arm to the home position (i.e., 600,000 μsteps, see the ESI Figure S3).
  g. Prime the MHQ mixer arm.
    i. Enter the program code /1H04M4500A50000A600000R into the LinControl command line; for an explanation of the code, see the note below.
    ii. To test the functionality of the mixer arm, press “execute” in the LinControl software; the mixer-arm is now primed.
    iii. Press “execute” again; now, the mixer-arm should move down into the cold-plate before returning to the home position. To prepare the mixer arm for sample preparation, press “execute” again; now the mixer-arm is again primed.
      ***Note:*** The command used by the LinControl software has the form: /1H04M4500A50000A600000R; where “/” indicates the start of the command string, “1” is the controller address, “H04” indicates the signal from the injector, “M4500” is the mixer-arm wait-time upon execution start and before moving the mixer, “A50000” triggers the movement to absolute position 50,000 μsteps (i.e., on the initial downward trajectory), “A600000” triggers the movement to absolute position 600,000 μsteps (i.e., on the return upward trajectory), and “R” initiates the program.
      **CRITICAL:** When priming the mixer-arm, the injection valves must be in the “load” position, otherwise the command will auto-execute whenever the “execute” button is clicked.
  h. Prime the cold-plate.
    i. In IndraWorks, enter the “optimization” tab and activate the rotor-lock. A warning panel will appear, indicating that the motor is primed and manual rotation of the cold-plate is inhibited.
    ii. Go to the “set point” tab, enter 5 rpm, and confirm that the cold-plate rotates. Set the cold-plate stationary again (0 rpm).
      **CRITICAL:** Do not adjust the rotation speed of the cold-plate with personnel nearby. Only set speeds >100 rpm (or specifically 7000 rpm) once the vacuum lid is securely placed and the vacuum has sealed (troubleshooting 4). Avoid placing objects atop the computer, as switching windows may inadvertently trigger rotation of the cold-plate.
27. Enter one of the program codes given in Table 4 (each corresponding to a specific mixer-arm wait-time) and press “execute” to prime the mixer-arm. Press “execute” again; the mixer-arm will move vertically into the cold-plate before returning to the home position. Press “execute” once more to ensure that the mixer-arm is primed and ready for sample acquisition.

***Note:*** The following procedure is ideally performed by two individuals, referred to as person A and person B. Table 5 summarizes their specific roles, with further details provided below (step 28). This experiment determines the optimal mixer-arm wait-time and should be performed at ambient temperature (18°C–25°C). Avoid cooling the cold-plate to cryogenic temperatures, as this can cause additional sample dilution due to moisture condensation on the cold-plate.

28. Conducting the methylene blue dye wait-time dilution series:
  a. Person A injects the methylene blue dye and MilliQ H_2_O into the sample loops, switches the injection valves to the “inject” position, and starts a 15-min timer.
  b. Person B collects the dye as it exits from the nozzle by holding a 15 mL Falcon tube in alignment with the top of the cold-plate.
    ***Note:*** The tube should follow the mixer-arm’s downward movement (outside the cold-plate), remaining positioned until the mixer-arm returns to a position above the cold-plate.
    ***Note:*** This experiment estimates the extent of sample dilution occurring during deposition inside the cold-plate during the shot. Because the alignment of the collection tube relies on visual inspection and may lack precision, it is recommended to record triplicate measurements for each time point.
  c. Person A transfers the collected methylene blue solution into an Eppendorf tube using a Pasteur pipette and measures the A_280nm_ in triplicate using a Nanodrop spectrophotometer.
  d. Person B primes the MHQ for the next sample:
    i. Wash the ligand syringe with 10–15 mL of MilliQ H_2_O.
    ii. Load 100 μL methylene blue and MilliQ H_2_O into the ligand and protein syringes, respectively, and switch the injection valve position to “load”.
    iii. Prime the mixer-arm with the program code corresponding to the next time point.
  e. When the timer ends, the system will have equilibrated back to MilliQ H_2_O (see step 22). To collect the next time point, repeat steps 27–28.
    ***Note:***Step 28e ensures the system is washed for approx. 10 min after each dye sample collection, thoroughly removing residual dye between different mixer-arm wait-time replicates.
29. For each mixer-arm wait-time (*t*_*w*_), determine the dilution factor by dividing the A_280nm_ of that time point by the A_280nm_ of the methylene blue stock solution.

***Note:*** The dilution profile can be approximated by a parabolic function, considering two limiting cases: (i) At very short mixer-arm wait-times ($\lim_{t\to 0}t_{w}\left(t\right)$; A_280nm_ = 0), the dye does not have time to move from the sample loop to the mixer. As a result, only MilliQ H_2_O is collected, and the A_280nm_ reading is zero. (ii) At very long mixer-arm wait-times ($\lim_{t\to \infty }t_{w}\left(t\right)$ ; A_280nm_= 0), all the dye is ejected above the cold-plate and ends up in the waste. Again, the A_280nm_ reading is zero.

30. Fit the measured dilution series using Equation 4:

(Equation 4) $$f\left(t_{w}\right)=\frac{V_{total}}{\left(V_{dye}-k\sqrt{{\left(t_{0}-t_{w}\right)}^{2}}\right)}$$

***Note:*** Here *V*_*total*_, the combined volume of methylene blue and MilliQ H_2_O injected into the sample loops; *V*_*dye*_, volume of methylene blue injected into the sample loops; *k*, flow rate of each channel in μL s^−1^; *t*_*w*_, the mixer-arm wait-time in s; *t*_0_, fit parameter, representing the hypothetical time at which only dye is collected (the symmetry axis of the parabola). Physically, *t*_0_ should be greater than the predicted optimal wait-time, as the minimum of the parabola is not a sharp point (Delta function) but has a measurable width.

***Note:*** In the above example, *V*_*total*_ is 200 μL, *V*_*dye*_ is 100 μL, *k* is 16.67 μL s^−1^, and *t*_0_ is 5.33 s. One can define the dead volume (*V*_*d*_) in each channel of the MHQ (i.e., the volume of the tubing from the sample loops to the mixer) using Equation 5:

(Equation 5) $$V_{d}=\pi r^{2}h$$

where *h* is the length of the tubing, with radius *r*. In our setup the tubing length is approximately 1.85 × 10^3^ mm, while the radius is 1.27 × 10^−1^ mm, corresponding to a dead-volume of approximately 94 μL (per channel). At a total (both channels combined) flow-rate (*f*) of 2 mL min^−1^ (i.e., 16.7 μL s^−1^ in each channel), the time to travel from the sample loops to the mixer (i.e., the quotient of the dead-volume and the flow-rate) $t_{s\to m}$ is 5.63 s. Nominally, the optimal mixer-arm wait-time before the vertical movement will be some period (*t*_*d*_; or mixer dead time, see Equation 7 below) shorter than 5.63 s post-injection, because the mixer-arm also must align the nozzle/jet to the top of the cold-plate. Solving for *t*_*d*_ requires determining the optimal mixer-arm velocity (*v*_*m*_), which depends on the total sample volume (*V*_*s*_) and the flow rate per channel, *f*, as defined in Equation 6:

(Equation 6) $$v_{m}=\frac{2d_{c}\times f}{V_{s}}$$

where *d*_*c*_ is the mixer-arm displacement within the cold-plate, which will depend on the vertical end position of the mixer within the cold-plate. In our instrument, *d*_*c*_ is approximately 49.6 mm (i.e., from the top of the cold-plate to the bottom), yielding a mixer-arm velocity of 16.5 mm *s*^−1^. For the step-motor controlling the mixer-arm and in the LinControl software, μstep is the unit of displacement, where 1 × 10^5^ μsteps correspond to a distance of 12.4 mm (Figure S3). Therefore, the optimal mixer-arm velocity is 1.33 × 10^5^ μsteps *s*^−1^. We can now determine *t*_*d*_ using Equation 7:

(Equation 7) $$t_{d}=\frac{d_{m\to c}}{v_{m}}$$

where $d_{m\to c}$ is the mixer-arm displacement to the top of the cold-plate, which will depend on the start position of the mixer above the cold-plate. In our instrument, $d_{m\to c}$ is approx. 18.6 mm, corresponding to 1.50 ×10^5^ μsteps and yielding a *t*_*d*_ of 1.13 s. The optimal mixer-arm wait-time (*t*_*w*_) can be compared to the theoretical value using Equation 8:

(Equation 8) $$t_{w}=t_{s\to m}-t_{d}$$

**Table 2:** Input parameters for the LinControl software

Parameter	Input value	SI units value
Top velocity	133,333 μsteps s^−1^	16.5 mm s^−1^
Home position	600,000 μsteps	74.4 mm
Acceleration	1,000 μsteps s^−2^	0.124 mm s^−2^
Move absolute	400,000 μsteps	49.6 mm

**Table 3:** Aging time as a function of the distance between the nozzle outlet and the cold-plate

Distance (Nozzle ↔ Cold-plate)/mm	Transport time *t*_*t*_/μs	Aging time *t*_*a*_/μs
3	47	82
5	66	99
7	85	116
10	113	142
17	179	201
23	236	252
29	292	303
41	405	405
52	509	498
61	594	574
72	697	668

**Table 4:** LinControl program codes for different mixer-arm wait-times

Program code	Mixer-arm wait-time/ms
/1H04M2500A50000A600000R	2500
/1H04M3500A50000A600000R	3500
/1H04M4500A50000A600000R	4500
/1H04M6500A50000A600000R	6500
/1H04M8500A50000A600000R	8500
/1H04M10500A50000A600000R	10500

**Table 5:** Roles of person A and person B during the acquisition of an MHQ wait-time dilution series

Time	Person A	Person B
Before timer start	Inject the methylene blue dye and MilliQ H_2_O into the sample loops; set a timer for 15 min.	Position a 15 mL Falcon tube in line with the top of the cold-plate and in front of the nozzle.
*t* = 0 min	Switch the injection valve positions from “load” to “inject”.	Observe for pressure spikes.
*t* when the mixer-arm aligns with the top of the cold-plate (downward trajectory)	-	Collect the sample into the Falcon tube.
*t* when the mixer-arm aligns with the top of cold-plate (upward trajectory)	-	Stop collecting the sample
*t* < 2 min	Transfer the sample into an Eppendorf tube with a Pasteur pipette.	Wash the ligand syringe with MilliQ H_2_O.
*t* = 5 min	Measure A_280nm_ via Nanodrop spectrophotometer in triplicate.	Switch the injection valve position to “load”; refill syringes.
*t* = 15 min	-	Prime the mixer-arm for the next time-point or replicate; stop the timer.

This allows modelling the behavior of the optimal mixer-arm wait-time as a function of flow-rate, sample volume, and aging time (see ESI Figure S4).

***Optional:*** To assess the dilution at different stages of MHQ sample handling (MHQ mixing, cold-plate deposition, and sample scraping and packing), it is recommended to perform a full complement of MHQ warm- and cold-shooting dilution series with methylene blue at least once.

***Note:*** The rationale of the following steps is to assess the dilution resulting from condensation of moisture (i) during sample deposition onto the cold-plate and (ii) during sample packing into an EPR tube.

31. Perform an MHQ “cold-shooting” series with methylene blue. As preparation, repeat steps 23–27, entering the program code corresponding to the empirically determined optimal wait-time.

***Note:*** The following steps outline the MHQ sample acquisition procedure of a single aging-time point, using MilliQ H_2_O and a methylene blue solution as mixing components. This protocol can be adapted for other systems, such as protein-ligand pairs.

32. Pour approx. 50 L of liquid nitrogen (N_2(l)_) into two 25 L standing Dewar flasks.
33. Fill a Styrofoam box with N_2(l)_ and submerge the following items:
  a. A plastic ladle for removing N_2(l)_ from the cold-plate during sample acquisition.
  b. A plastic spoon for transferring samples from the cold-plate.
  c. A wooden scraper for detaching samples from the walls of the cold-plate.
  d. The MHQ exhaust tubing; after vacuum ventilation under the MHQ lid, cold nitrogen gas will restore atmospheric pressure.
34. Insert an EPR tube (3 mm o.d. for Q-band, 4 mm o.d. for X-band measurements) into the funnel apparatus (see ESI, Figure S3).

**CRITICAL:** The funnel has different adapters for X- and Q-band EPR tubes, make sure to choose the adapter with the correct aperture.

Tighten the adapter by hand and ensure the tube is held in place tightly; insert a packing rod and gently press it to the bottom of the tube several times.

***Note:*** Slight resistance without slippage indicates proper mounting. For X-band measurements, shorten the tube to approx. 21 cm so that it fits inside the Dewar flask when mounted.

**Pause point:** At this stage, the MHQ is equilibrated, and the mixer-arm is primed. The MHQ can remain in this state for up to 12 h before exchanging back into MilliQ H_2_O and then 20% (v/v) EtOH.

35. Prepare pre-cooled isopentane:
  **CRITICAL:** Always wear appropriate PPE when handling isopentane and N_2(l)_. At a minimum, use insulated thermal gloves and safety goggles; a face shield is recommended.
  a. In a flat-bottom metal container (15 cm height, 15 cm i.d., approx. 2.7 L volume, with lid), place a magnetic stirring rod, a digital thermometer, and approx. 1 L isopentane.
  b. Set the metal container in a Styrofoam box on a magnetic stirrer plate and stir at approx. 400 rpm.
  c. Fill the Styrofoam box with N_2(l)_ to partially submerge the metal container. Continue adding N_2(l)_ until the temperature of the isopentane reaches approx. −150°C (do not go lower, as isopentane will freeze).
  d. Transfer the pre-cooled isopentane to a metal Dewar flask equipped with a magnetic stirring rod and a digital thermometer. Immerse the EPR tube (mounted on the funnel) into the isopentane.
  e. During transfer, the isopentane may warm slightly. Gradually add small amounts (approx. 5–10 mL) of N_2(l)_ to maintain a temperature of ≤ −140°C, compensating for minor warming during sample preparation and packing.
    **CRITICAL:** Keep isopentane at temperatures ≤ −130°C to prevent sample thawing during packing.
36. Pre-cool the sample funnel:
  a. Place a bunched towel over the inner funnel to minimize moisture condensation inside the EPR tube during cooling.
  b. Gradually cool the outer funnel with N_2(l)_ by incrementally adding N_2(l)_, ensuring that none spills into the inner funnel. Continue this process for approx. 20 min. At the end of this period, N_2(l)_ should remain in the outer funnel without exhibiting nucleative boiling.
  c. Place a second towel over the outer funnel to reduce moisture condensation from the air and to slow down nitrogen evaporation.
37. Prepare the pre-cooled cold-plate:
  a. Start the MHQ device and set cold-plate rotating.
    i. Cover the cold-plate with a towel and begin rotating at 5 rpm (see step 26h).
    ii. Start the internal heat gun.
    iii. Activate both HPLC pumps (for the protein and the ligand channels), and set each pump to a flow rate of 1 mL min^−1^.
      ***Note:*** The internal heat gun prevents the cold-plate rotor from freezing and avoids buffer freezing, which can block the HPLC pumps and cause pressures to exceed 6000 psi. Maintaining buffer flow through the MHQ during the entire cooling of the cold-plate also helps prevent buffer freezing in the tubing. Troubleshooting 1.
  b. Fill the cold-plate with approx. 2.5 L N_2(l)_ and set a timer for 20 min.
    **CRITICAL:** Ensure the cold-plate remains continuously submerged in N_2(l)_ throughout this step. Do not allow the inside of the cold-plate to run dry at any point during the cool-down.
  c. After 20 min, refill the cold-plate with N_2(l)_ one final time. The cold-plate is now ready for MHQ sample acquisition.
    ***Note:***Steps 38–40 can be performed while waiting for step 37b to complete.
38. Purge the Hamilton syringes for protein and ligand with MilliQ H_2_O.
  a. Draw 100 μL each of methylene blue solution and MilliQ H_2_O into the syringes, ensuring all air bubbles are removed.
  b. Insert the syringes into the MHQ injection valves in the load position.
39. Confirm that the MHQ ventilation valve is closed. Open the valve and verify that the vacuum pump is drawing suction by listening for air movement through the vents.

***Optional:*** To check if the mixer-arm is primed, shift the protein injection valve to “inject”; after approx. 5 s, the mixer-arm should begin moving downward. Return the protein injection valve to “load” and press “execute” again.

40. Prepare Dewar flasks and packing rods.
  a. Fill two Dewar flasks completely (approx. 2 L each) with N_2(l)_, and a third flask halfway (approx. 1 L).
  b. Place the packing rods into one of the full Dewar flasks.
  c. Cover all Dewar flasks with dry towels.
  d. Remove the waste collector from the MHQ.

***Note:*** The second full Dewar flask is used to refill the cold-plate after ventilation and re-establishment of atmospheric pressure under the MHQ lid. The half-full Dewar flask is used for decanting the sample from the cold-plate and allowing sample particles to sediment.

41. Acquire the MHQ sample. Troubleshooting 1, troubleshooting 4, troubleshooting 5.
  ***Note:*** This step requires two operators, referred to as person A and person B. Table 6 summarizes their specific roles. Both must wear PPE, including a face shield and cryoprotection gloves.
  **CRITICAL:** Person A must wear insulated thermal gloves and a face shield during step 41a. The sample should be transferred into N_2(l)_ within approx. 2 min of starting step 41a.
  a. Person A empties the cold-plate of N_2(l)_ using the ladle, quickly wipes the cold-plate with a towel (1–2 s), and positions the mixer-arm over the cold-plate. Person B places the MHQ lid, while person A secures it using the fastening tips.
  b. Person A opens the vacuum valve while person B firmly holds down the lid. Monitor the pressure under the vacuum lid; it should begin to decrease.
  c. If the pressure is decreasing, person A injects the contents of the Hamilton syringes into the sample loops. In the IndraWorks software, enter a value of 7000 rpm and execute when the pressure under the MHQ lid has reached 100 mbar.
  d. When the pressure under the MHQ lid reaches 80 mbar, person A switches the ligand injection valve to “inject”, waits 1 s, then switches the protein injection valve to “inject”.
  e. As the mixer-arm begins its downward movement into the cold-plate, person A enters 20 rpm in the IndraWorks software and executes when the mixer arm returns to the home position.
    **CRITICAL:** During steps 41c–41e, person B must monitor the pressure of the HPLC pumps, ensuring it stays below 2000 psi. Person B should also monitor the sample loop connectors for leaks.
  f. Person A closes the vacuum valve while person B vents the MHQ and removes the lid. Person A fills the cold-plate with approx. 2.5 L of N_2(l)_. Person B returns the waste collector to the MHQ.
    **CRITICAL:** Do not overfill the cold-plate, as sample particles may escape when scraped from the cold-plate wall.
  g. Using the cooled wooden scraper, person A scrapes the sample from the cold-plate wall. The sample is deposited in layers on the cold-plate wall; adjust the height of the wooden scraper as needed for efficient removal of the sample.
    ***Note:*** To calculate the number of layers (*N*_*layers*_), the MHQ jet can be modelled as a cylinder using Equation 9:
    (Equation 9) $$l_{jet}=\frac{V_{s}}{{\pi r_{jet}}^{2}}$$
    where *l*_*jet*_ is the length that the MHQ jet would have if it were to accommodate the whole sample volume (*V*_*s*_) at once, and *r*_*jet*_ is the radius of the jet (i.e., of the orifice).
    The inner circumference of the cold-plate (*U*) is given by Equation 10:
    (Equation 10) $$U=2\pi r_{cold-plate}$$
    where *r*_*cold*-*plate*_ is the inner radius of the cold-plate (herein *r*_*cold*-*plate*_= 6.5 × 10^1^ mm, and therefore *U*= 4.08 × 10^2^ mm). The speed of deposition (*v*_*deposition*_) of the jet onto the cold-plate is given by Equation 11:
    (Equation 11) $$v_{deposition}=v_{cold-plate}\times U$$
    where *v*_*cold*-*plate*_ is the speed of rotation of the cold-plate in revolutions s^−1^, and *U* is defined above in Equation 10. The theoretical time taken to deposit the sample (*t*_*deposition*,*theory*_) as a single layer onto the cold-plate is then calculated using Equation 12:
    (Equation 12) $$t_{deposition,theory}=\frac{l_{jet}}{v_{deposition}}$$
    The experimental time taken to deposit the sample (*t*_*deposition*,*sample*_) is calculated using Equation 13:
    (13) $$t_{deposition,sample}=\frac{V_{s}}{2f}$$
    Finally, *N*_*layers*_ can be calculated using Equation 14:
    (Equation 14) $$N_{layers}=\frac{t_{deposition,theory}}{t_{deposition,sample}}$$
    As the number of layers increases, the cryofixation time at the cold-plate surface will become more heterogeneous, so the number of layers should be kept close to unity (for example, in this work 2 <*N*_*layers*_< 3).
    To calculate the thickness of a single sample layer, it is first necessary to calculate the area of the cold-plate coated in sample; this forms a rectangle of length *U* (defined above) and the height *d*_*c*_ (i.e., the mixer-arm displacement within the cold-plate). The sample film can then be represented as a cuboid of the width *x*, defined using Equation 15:
    (Equation 15) $$x=\frac{V_{s}}{U\times d_{c}}$$
    For a sample volume (*V*_*s*_ = 200 μL) and a cold-plate with dimensions similar to our instrument (*U* = 4.08 × 10^2^ mm and *d*_*c*_ = 4.34 × 10^2^ mm), a sample thickness of approximately 11 μm is obtained (for approximately 2 layers). This corresponds to a thickness of approximately 5.5 μm per layer. These equations allow modelling of the number of sample layers as a function of (i) flow-rate and (ii) sample volume. Additionally, the thickness of sample layers can be modelled as a function of sample volume (see ESI Figure S5).
42. Using a cooled plastic spoon, transfer the sample particles into the Dewar flask half-filled with N_2(l)_. Cover the Dewar flask with a dry towel and allow the sample particles to settle to the bottom (approx. 10–15 min).

***Optional:*** Pour some N_2(l)_ from the Dewar flask back into the cold-plate. Repeat steps 41g and 42 several times to maximize sample recovery and keep the sample particles frozen.

***Note:***Steps 43 and 44 can be performed while waiting for step 42 to complete, see below.

43. Warming up the cold-plate.
  a. Heat the cold-plate with an external heat gun directed into the cold-plate. Set a timer for 13 min.
  b. After the time has elapsed, switch off the heat gun, clean the cold-plate with MilliQ H_2_O, and dry it with a clean towel.
  c. Stop the cold-plate rotation by entering a value of 0 rpm.

**CRITICAL:** Wear insulating thermal gloves and a face shield during step 43, as temperatures can exceed 570°C while using the heat gun.

44. Thaw the MHQ sample. Determine the dilution factor of the methylene blue collected from the cold-plate relative to the stock solution using UV-visible spectrophotometry.
  ***Note:*** Execution of step 44 depends on whether the dilution (i) only from sample deposition onto the cold-plate or (ii) from sample deposition and packing into an EPR tube is being investigated. Triplicate measurements are desirable.
  a. To assess the dilution from sample deposition onto the cold-plate only, transfer the sample to a 15 mL Falcon tube. Thaw and proceed with step 47.
  b. To assess the dilution from both sample deposition and packing into an EPR tube, continue with steps 45 and 46. After packing, thaw the sample in the EPR tube and continue with step 47.
45. Pack the collected MHQ sample into an EPR tube. Troubleshooting 2**.**
  ***Note:*** Refer to ESI Figure S3 for details on the funnel apparatus used to transfer and compress the sample in the EPR tube.
  a. Gradually decant approx. 80% of the excess N_2(l)_ from the Dewar flask containing the sample, pausing periodically to allow agitated particles to settle.
    ***Note:*** Be careful not to spill sample particles. The N_2(l)_ is translucent, whereas the sample particles are colored.
  b. Agitate the sample and quickly (1–2 s) decant the remaining N_2(l)_ into the inner funnel (see ESI Figure S3).
    ***Note:*** Perform this decanting step in a single motion to avoid the deposition of sample particles on the walls of the Dewar flask.
  c. Allow the sample particles to settle (approx. 1–2 min) in the inner funnel. Keep the outer funnel approx. 75% full of N_2(l)_, avoiding spillage into the inner funnel and the EPR tube.
    **CRITICAL:** When topping up the N_2(l)_ in the outer funnel, it is important to do so gradually; topping up too quickly can cause large amounts of water ice to form, potentially blocking the EPR tube.
  d. Insert the packing rod through the inner funnel and gently push it to the bottom of the EPR tube in a twisting motion. Retract the rod halfway, then repeat the process.
    ***Note:*** This ensures that any sample particles adhering to the tube walls are pressed down to the bottom.
    **CRITICAL:** Prevent the packing rod from warming during this step. Routinely submerge the rod in N_2(l)_ and consider alternating between two rods to maintain low temperature.
  e. Use the packing rod to gently stir the N_2(l)_ in the inner funnel. This helps prevent sample particles from settling on the walls of the inner funnel.
  f. Repeat steps 45d and 45e until all the N_2(l)_ in the inner funnel has evaporated (approx. 10–15 min). As the N_2(l)_ evaporates from the inner funnel, you may feel slight resistance with the packing rod, indicating the formation of a slurry of sample particles.
46. Dismounting the sample tube:
  a. Slightly lift the funnel out of the isopentane bath to expose the connector between the funnel mouth and the EPR tube.
  b. Using a pair of tweezers and a small rubber bulb, position the EPR tube between the tweezers, resting them across the top of the Dewar flask and below the connector.
    ***Note:*** This setup prevents the connector from falling into the isopentane bath and damaging the EPR tube during removal.
  c. Unscrew the connector by hand or with a pair of wrenches.
    ***Note:*** If the connector is frozen in place, use a hair dryer to thaw it before unscrewing. Take care not to thaw the sample inside the EPR tube.
    **CRITICAL:** Never point the hair dryer directly into the Dewar flask, as it contains highly flammable isopentane.
  d. Once dismounted, remove the tube from the isopentane bath and quickly (within 1–2 s) wipe off any residual isopentane from the tube surface. Immediately submerge the sample tube in N_2(l)_ and label it. Store the tube in N_2(l)_ until further use.
    ***Note:*** The sample height in the EPR tube should be approx. 2–4 cm – enough to fill the active volume of the EPR resonator. It may vary depending on relative humidity and moisture condensation either onto the cold-plate or during packing.
    **Pause point:** If you need to prepare another sample, repeat the protocol from step 32. If sample acquisition is complete for the day, proceed to step 48.
47. Determine the dilution of the methylene blue after MHQ.
  a. Transfer the thawed sample to a Nanodrop spectrophotometer using a Pasteur pipette.
  b. Measure the A_280nm_ for the collected samples. Calculate the dilution by dividing the A_280nm_ of the sample by the A_280nm_ of the methylene blue stock solution measured earlier in step 23d.

***Note:*** In our experiments, the aggregate dilution effects – resulting from mixing, deposition onto the cold-plate, and sample packing – were highly consistent with prior literature.^14^ For a detailed discussion of individual contributions to dilution during MHQ sample acquisition, refer to the expected outcomes and Figure 2.

48. MHQ storage after sample preparation.
  a. Switch the protein and ligand injection valves from “inject” to “load”.
  b. Reduce the flow rate on each HPLC pump to 0 mL min^-1^. Wait until the pressure drops to 0 psi. Remove the HPLC tubing from 1 × PBS buffer (see step 9a) and immerse it in filtered MilliQ H_2_O.
  c. Reset the flow rate to 1 mL min^-1^ at each pump. Ensure that the protein and ligand sample injection valves both remain in the “load” position.
  d. After 15 min, switch both sample injection valves to the “inject” position. Monitor the pressure.
    ***Note:*** A pressure increase indicates air bubbles or debris in the tubing; a pressure decrease indicates a leak.
  e. After 5 min, switch both sample injection valves back to the “load” position. Reduce the flow rate to 0 mL min^-1^ at each pump. Wait for the pressure to return to 0 psi.
  f. Repeat step 22 with filtered 20% (v/v) EtOH. Switch off the HPLC pumps.
  g. Close the LinControl and IndraWorks software.
  h. Switch off the MHQ device.
49. For calibration of MHQ aging times, prepare solutions of horse heart metmyoglobin (MetMb) and sodium azide (NaN_3_):
  **CRITICAL:** Prepare and pH-adjust the NaN_3_ solution in a fume hood. Acidification of concentrated NaN_3_ solution can produce HN_3_, a highly toxic gas. Always wear appropriate PPE (safety goggles and nitrile gloves).
  ***Note:*** Release of HN_3_ reduces the concentration of the N_3_^-^ anion, potentially lowering the concentration of the stock solution after pH adjustment. Additionally, the rate constant of azide binding to metmyoglobin increases as pH decreases.^2^
  a. Vacuum-filter 1 L of citrate buffer (25 mM sodium citrate, pH 5) using a 0.45 μm filter.
  b. Weigh 26.0 mg of MetMb (molecular weight: 16,951 Da)^15^ and dissolve in 1 mL of filtered citrate buffer (final concentration: 1.5 mM).
  c. Centrifuge the MetMb solution (13,000 × *g*, 5 min, 4°C) to remove precipitates. Transfer the supernatant to a fresh Eppendorf tube and store on ice until use.
    ***Note:*** Prepare fresh MetMb stocks every 24 h.
  d. Prepare sodium azide stock. Weigh 487.6 mg of NaN_3_ (molar mass: 65.01 g mol^-1^) and dissolve in 5 mL of filtered citrate buffer (final concentration: 1.5 M).
  e. Filter the NaN_3_ stock solution through a 0.45 μm syringe filter and store on ice until use.
50. Prepare the MHQ device for sample acquisition.
  a. Ensure that 1-mL sample loops are installed in the MHQ device.
    ***Note:*** For calibration with MetMb, use tubing volumes of 500 μL in the protein channel and 1 mL in the ligand channel to ensure sufficient sample for X-band CW-EPR spectroscopy.
    ***Optional:*** When connecting the sample loops, use a wrench to tighten them beyond finger-tight, as high pressure during injection may cause sample loops to eject unexpectedly.
    **CRITICAL:** After replacing sample loops, check for leaks by performing several mock injections of citrate buffer into the protein and the ligand channel. Switch the injection valve position from “load” to “inject”. Robust connections should withstand pressures of over 2000 psi without leakage.
  b. Repeat steps 22, 24, 26a-c, and step 26e.
  c. Enter a program code into the LinControl software to control vertical movement of the mixer-arm. Refer to steps 26d and 27 for details.
    i. Calculate the mixer-arm’s optimal wait-time for a total volume of 1.4 mL, and execute the program/1H04MXXXXA50000A600000R (where XXXX is the theoretical optimal mixer-arm wait-time in milliseconds).
      ***Note:*** For aging-time calibration, use a mixer-arm wait-time of 8.5 s. Note that some sample may be lost to the waste rather than deposited onto the cold-plate. However, sample availability is ample: 1.4 mL is approximately seven times the active volume of the resonator.
    ii. Confirm the mixer-arm is primed by switching the injection valve from “inject” to “load”. The mixer-arm should descend into the cold-plate.
  d. Load 1 mL of NaN_3_ solution into the ligand syringe and 400 μL of MetMb solution into the protein syringe, then insert the syringes into the injection valves.
  e. Mount an X-band EPR tube (4 mm o.d.) into the MHQ funnel (see step 34).
51. Repeat step 32 and 33.
52. Repeat steps 35-43, step 45, and step 46. Troubleshooting 2, Troubleshooting 4**.**
53. CW-EPR spectroscopy of MetMb samples at 20 K.
  ***Note:*** Use the EMXmicro EPR spectrometer with the ER4122SHQE resonator, the ER4112HV low-temperature control system, and the Mercury iTC503 temperature controller (sensor type ESR900). For details, refer to the user manual of the spectrometer and the accessories.
  a. Ensure sufficient supply of N_2(l)_ (approx. 1 L) to store the samples and of liquid helium (approx. 100 L) for spectrometer cryostat cooling.
  b. Ensure the sample is densely packed in the EPR tube by the following steps.
    i. Prepare a cold bath from isopentane and liquid nitrogen (see step 35).
    ii. Transfer the sample tube into the cold isopentane within 2-3 s and anneal for approximately 10 min.
      ***Note:*** This procedure allows trapped N_2(l)_ in the sample to expand and boil off gradually, minimizing violent sample ejection when inserting the tube into the spectrometer. Troubleshooting 2.
    iii. Cool a sample packing rod in N_2(l)_. Carefully repack the sample to ensure all N_2(l)_ is removed from the tube. Briefly hold the tube above the isopentane surface and check for any off-gassing.
    iv. Fill a small Dewar flask (e.g., Type 00C from KGW Isotherm, 13.5 cm outer height) with N_2(l)_. Remove the sample tube from the isopentane, quickly wipe off any residual isopentane from the tube’s surface, and immerse the tube into N_2(l)_.
      **CRITICAL:** The sample must be densely packed in the tube and completely free of N_2(l)_. Any remaining N_2(l)_ in the sample powder can expand during the transfer of the EPR tube from the Dewar flask to the cryostat, potentially causing violent ejection of the sample or tube explosion if venting is blocked. Troubleshooting 2.
  c. Cool the spectrometer’s cryostat to 20 K.
    **CRITICAL:** Always wear PPE during the following steps:
    i. Connect the turbomolecular pump to the cryostat and switch it on. Ensure the pressure inside the cryostat reaches ∼10^-4^ mbar before continuing.
    ii. Attach the overflow valve of the helium tank to a helium recovery system (if available) and ensure that gas flow within the tubing is not obstructed (e.g., due to bending).
      ***Note:*** Leave the overflow valve open to prevent overpressure in the tank.
    iii. Open the needle valve on the transfer line. Carefully insert the transfer line into the helium tank over approximately 2 min; secure it with a spanner once fully inserted.
      ***Note:*** Monitor the tank pressure during insertion and ensure it does not exceed the safety threshold indicated on the manometer.
    iv. Confirm gas flow by momentarily immersing the outlet of the transfer line in ethanol, then immediately wipe it and connect it to the cryostat of the spectrometer.
    v. Connect the transfer line with the helium recovery system (if available).
    vi. Connect the membrane pump to the transfer line. Close the cryostat with a cap to prevent nitrogen condensation during cooling.
    vii. Open the needle valve of the transfer line as needed (approx. ¼ turn) and start the membrane pump to establish a steady flow of cold helium gas (approx. 1 L h^-1^).
      ***Note:*** Cooling the cryostat from 298 K to 20 K usually takes 20–30 min. If necessary, adjust the flow rate by fine-tuning the needle valve at the transfer line: a flow rate that is too high may cause temperature fluctuations, whereas too low a rate may prevent reaching the target temperature.
  d. Inserting the sample into the spectrometer.
    i. Switch off the membrane pump that transfers cold helium gas and allow the cryostat pressure to return to ambient levels.
    ii. Remove the EPR tube from the Dewar flask, quickly wipe it with soft tissue, and insert it into the cryostat.
      ***Note:*** Complete this procedure within 5–10 s to avoid sample warming.
    iii. Immediately restart the membrane pump and wait 5-10 min until the temperature reaches and stabilizes at 20 K.
      ***Note:*** To minimize the tube’s exposure to ambient temperature, position the Dewar flask as closely as possible to the spectrometer, ideally between the magnet coils.
      **CRITICAL:** Set the magnetic field of the spectrometer to 0 G before moving the Dewar flask between the solenoid coils; Dewar flasks are magnetic and may be attracted by the field.
  e. Record the CW-EPR spectrum using the parameters given in Table 7. For further details, consult the user manual.
54. Measure the signal intensities for peaks associated with both *high*-spin and *low*-spin Fe(III) and calculate the proportion of the *apo*-state in the sample using Equation 16:

(Equation 16) $$\%{hs}_{apo}=\frac{I\left({hs}_{apo}\right)}{I\left({hs}_{apo}\right)+NF\times I\left({ls}_{holo}\right)}\times 100$$

***Note:****I*(*hs*_*apo*_) is the peak-to-peak amplitude of the perpendicular component in the *high*-spin Fe(III) spectrum (g_⊥_ = g_xx_ = g_yy_ = 5.8) and *I*(*ls*_*holo*_) is the peak-to-peak amplitude of the g_yy_-component in the *low*-spin Fe(III) spectrum (g_yy_ = 2.2). NF is a normalization factor to account for different intensities between *high*-spin and *low*-spin Fe(III) signals for nominally identical numbers of spins.^16^^,^^17^ The intensity difference mostly arises from the different relaxation times and saturation behaviors of *high*-spin and *low*-spin Fe(III). NF is given by Equation 17^16^^,^^18^:

(Equation 17) $$NF=\frac{I\left({hs}_{apo}\right)}{I\left({ls}_{holo}\right)}$$

***Note:*** A normalization factor (NF) of 15.49 was obtained under the experimental conditions described above. This implies considerably faster electron-spin relaxation of the *high*-spin Fe(III) component, as the *low*-spin Fe(III) component saturates more readily. This is consistent with the literature.^19^

55. Fit a bi-exponential decay function to the *apo*-state fraction, *f*_*apo*_, as a function of the aging time (see Table 3) using Equation 18:

(Equation 18) $$f_{apo}=A\times \exp\left(-k_{1}^{\prime }t_{a}\right)+B\times \exp\left(-k_{2}^{\prime }t_{a}\right)$$

***Note:*** Here, *f*_*apo*_ is the *apo*-state fraction, *A* and *B* are pre-exponential coefficients, $k_{1}^{\prime }$ and $k_{2}^{\prime }$ are pseudo-first order rate constants, and *t*_*a*_ the aging time.

***Note:*** Pseudo-first order kinetics is a fair approximation here, due to a 1000-fold excess of NaN_3_ relative to MetMb. The reaction is assumed to proceed in absence of intermediates; therefore, the state fractions (i.e., *apo* and *holo*) always sum to unity.

56. Compare the extracted rate constants to published values.^2^ Ideally, the data should agree within ±10% with the literature.

***Note:*** Differences in pH may explain minor deviations from literature values, as the rate of azide binding increases as pH decreases.^2^^,^^20^

**Table 6:** Roles during MHQ sample acquisition for person A and B

Time/min	Person A	Person B
Before timer start	Remove the remaining N_2(l)_ from the cold-plate using a ladle and a towel; move the mixer-arm into position.	Remove the covering towel from the cold-plate.
*t* = 0	Put the fastening tips down onto the vacuum lid. Inject the protein and the ligand from the syringes into the sample loops. Start the vacuum pump.	Put the vacuum lid on the cold-plate and hold it down tightly. Start the timer.
*t* when pressure = 100 mbar	Start the rotor at 7000 rpm.	Observe for pressure spikes.
*t* when pressure = 80 mbar	Switch the valve position from “load” to “inject”.	Observe for pressure spikes and leakage.
*t* when the mixer-arm returns to home position	Set the rotor to 20 rpm.	Release the vacuum.
*t* when the mixer-arm returns to home position	Lift the fastening tips to unlock the lid.	Lift the lid.
*t* < 2 min	Move the mixer-arm away from the cold-plate. Fill the cold-plate with N_2(l)_. Scrape the sample from the cold-plate with a wooden scraper.	Stop the timer.

**Table 7:** Parameters for the CW*-*EPR measurement on the MetMb/NaN_3_ samples at 20 K

Parameter	Value
# of scans	5
# of points	3600
Center Field	2600 G (corresponding to g∼2.58)
Attenuation	25 dB (≙ 0.5 mW)
Sweep Width	4000 G
Receiver Gain	30 dB
Conversion Time	85 ms
Time Constant	81.92 ms
Modulation Amplitude	10 G

## Key resources table

**Table:** 

REAGENT or RESOURCE	SOURCE	IDENTIFIER
**Bacterial and virus strains**		

BL21(DE3) competent cells	New England BioLabs GmbH	Cat#C2527H
TOP10 competent cells	Thermo Fisher Scientific	Cat#C404003

**Chemicals, peptides, and recombinant proteins**		

Agar	ITW Reagents	Cat#402302
Ammonium peroxydisulfate	Carl Roth GmbH + Co. KG	Cat#9592.1 PubChem SID: 481106891
Ampicillin sodium salt	Carl Roth GmbH + Co. KG	Cat#K029.2 PubChem SID: 483924476
L-Arginine	Carl Roth GmbH + Co. KG	Cat#3144.2 PubChem SID: 505555665
Cyclic adenosine monophosphate (cAMP)	Sigma Aldrich	Cat#A9501 PubChem SID: 24278245
Deuterium oxide (D_2_O)	Deutero GmbH	Cat#00506-100ml PubChem SID: 10535810
Dimethylsulfoxide (DMSO)	Sigma Aldrich	Cat#D2438 PubChem SID: 24893688
Dithiothreitol (DTT)	Sigma Aldrich	Cat#43816 PubChem SID: 57650196
Equine heart metmyoglobin	Sigma Aldrich	Cat#M1882-250MG PubChem SID: 135274540
Ethanol (EtOH)	Thermo Fisher Scientific	Cat#365780025 PubChem SID: 481101440
Ethylenediamine tetraacetic acid (EDTA)	Carl Roth GmbH + Co. KG	Cat#8040.2 PubChem SID: 481107126
Ethylene glycol-d_6_	Deutero GmbH	Cat#15102 PubChem SID: 505386886
L-Glutamic acid	Carl Roth GmbH + Co. KG	Cat#3774.2 PubChem SID: 481107778
Guanidine HCl	Carl Roth GmbH + Co. KG	Cat#0035.1 PubChem SID: 483928925
Isopropyl-ß-D-thiogalactopyranoside (IPTG)	Sigma Aldrich	Cat#I6758-1G PubChem SID: 24896093
{2,2,5,5-Tetramethyl-3-[(2-methyl-2,2-dioxo-2λ^6^-disulfan-1-yl)methyl]-2,5-dihydro-1H-pyrrol-1-yl}oxylmethanethiosulfonate (MTSSL)	Biozol	Cat#MBS6063855-50 PubChem SID: 500711556
2-Methylbutane (isopentane)	Carl Roth GmbH + Co. KG	Cat#3927.2 PubChem SID: 481107258
Methylene blue	Carl Roth GmbH + Co. KG	Cat#A514.3 PubChem SID: 481107806
Potassium chloride (KCl)	Carl Roth GmbH + Co. KG	Cat#1LCT.2 PubChem SID: 481108010
Potassium dihydrogenphosphate (KH_2_PO_4_)	Carl Roth GmbH + Co. KG	Cat#P018.2 PubChem SID: 505814994
Protease inhibitor cocktail (EDTA-free)	Sigma Aldrich	Cat#11873580001
Sodium azide (NaN_3_)	Sigma Aldrich	Cat#769320-100G PubChem SID: 329767440
Sodium chloride (NaCl)	Thermo Fisher Scientific	Cat#447300010 PubChem SID: 481108026
Sodium citrate	Carl Roth GmbH + Co. KG	Cat#3580.4 PubChem SID: 481107675
Sodium dihydrogen phosphate (NaH_2_PO_4_)	Carl Roth GmbH + Co. KG	Cat#1H52.3 PubChem SID: 516572007
Sodium hydrogen phosphate (Na_2_HPO_4_)	Carl Roth GmbH + Co. KG	Cat#X987.1 PubChem SID: 505883730
Sodium phosphate (Na_3_PO_4_)	Carl Roth GmbH + Co. KG	Cat#T107.1 PubChem SID: 516579449
Sodium dodecyl sulfate (SDS)	Thermo Fisher Scientific	Cat#15525017 PubChem SID: 505883583
Tryptone	Carl Roth GmbH + Co. KG	Cat#8952.2 PubChem SID: 135283230
Yeast extract	Carl Roth GmbH + Co. KG	Cat#2904.3 PubChem SID: 481192129

**Critical commercial assays**		

Amylose resin	New England BioLabs GmbH	Cat#E8021S
HiLoad 16/60 Superdex 200 pg column	Cytiva	Cat#28989335
QIAprep spin miniprep kit	Qiagen	Cat#27104

**Deposited data**		

CNBD pulse dipolar EPR spectroscopy data	Hett et al.^1^	https://doi.org/10.1021/jacs.1c01081
Metmyoglobin continuous-wave EPR data	Hett et al.^1^	https://doi.org/10.1021/jacs.1c01081

**Oligonucleotides**		

Primer: CNBD I340C Forward GAGATCGCGGAATGCTTCCGCAAGACCG	This work	https://doi.org/10.1016/j.xpro.2026.104405
Primer: CNBD I340C Reverse CGGTCTTGCGGAAGCATTCCGCGATCTC	This work	https://doi.org/10.1016/j.xpro.2026.104405
Primer: CNBD E289C Forward CGAATCCGGTGTGCCTTGGCCCTGGCGCC	This work	https://doi.org/10.1016/j.xpro.2026.104405
Primer: CNBD E289C Reverse CAGGGCCAAGGCACACCGGATTCGGCGTC	This work	https://doi.org/10.1016/j.xpro.2026.104405
Primer: CNBD C263S Forward CGCCGTGATCAGCCGCATTGGCGAGC	This work	https://doi.org/10.1016/j.xpro.2026.104405
Primer: CNBD C263S Reverse CTCGCCAATGCGGCTGATCACGGCGCCC	This work	https://doi.org/10.1016/j.xpro.2026.104405
Primer: CNBD C331L Forward CCAGATGTTGCTCAGCAGCAGCCCGG	This work	https://doi.org/10.1016/j.xpro.2026.104405
Primer: CNBD C331L Reverse GGGCTGCTGCTGAGCAACATCTGGAAATC	This work	https://doi.org/10.1016/j.xpro.2026.104405

**Recombinant DNA**		

Plasmid: pMalc2X-CNBD-263S/331L	Hett et al.^1^	https://doi.org/10.1021/jacs.1c01081

**Software and algorithms**		

AUTODESK Viewer	AUTODESK	https://viewer.autodesk.com
DeerAnalysis 2019	Jeschke et al.^21^	https://epr.ethz.ch/software/older-versions/old_deeranalysis.html
Expasy ProtParam	Gill et al.^12^	https://web.expasy.org/protparam/
IndraWorks Version 8.6.172.0	Bosch Rexroth	www.boschrexroth.com/en/us/media-details/d7b57806-1b09-493f-a5ef-a7f970c2dccb
LinControl Version 1.0.1	Lin Engineering	https://www.linengineering.com
MATLAB 2019b	The MathWorks	https://www.mathworks.com/downloads/web_downloads/download_release?release=R2019b
OriginPro 8G	OriginLab	www.originlab.com
ProDEL Script “SafeAfterEachScan.ppp”	Github	https://github.com/TobiasHett/EPR_Apps
Xenon Software EMXnano: Version Xenon_nano 1.6 EMXmicro: Version Xenon 1.1b.108	Bruker BioSpin GmbH & Co. KG	https://www.bruker.com/en/products-and-solutions/mr/epr-instruments/epr-software/xenon.html
Xepr Software version 2.7	Bruker BioSpin GmbH & Co. KG	https://www.bruker.com/en/products-and-solutions/mr/epr-instruments/epr-research-instruments/xepr-software.html

**Other**		

10 μL Disposable Capillaries	Hirschmann Laborgeräte GmbH & Co. KG (vendor: Carl Roth GmbH & Co. KG)	Capillary pipettes, minicaps 10 μL Cat#L925.2
515 HPLC pump	Waters	Cat#WAT068980
5810 R Centrifuge	Eppendorf AG	Cat#5811000061
7725i Injection Valve	IDEX Health and Science	Cat#7725I
ÄKTA go Purifier Chromatograph	GE Healthcare	Cat#29383015
Aluminium Foil	Carl Roth GmbH & Co. KG	Cat#1399.1
Amicon Centrifugal Filter Unit (30 kDa M.W.C.O.)	Merck	Cat#UFC903008
Autoclave Laboklav 80-B 80 L Benchtop	New England Laboratory Supplies	Cat#481-0417
Dewar Flasks	KGW Isotherm	Type 00 C Cat#1021 Type 3 C Cat#1025
EMXmicro EPR spectrometer with ER4122SHQE resonant cavity and ER4112HV low-temperature control system	Bruker BioSpin GmbH & Co. KG	https://www.bruker.com/en/products-and-solutions/mr/epr-instruments/emx-micro.html
EMXnano EPR Spectrometer (or equivalent)	Bruker BioSpin GmbH & Co. KG	https://www.bruker.com/en/products-and-solutions/mr/epr-instruments.html
Fermenter	Infors HT	www.infors-ht.com/en/products/bioreactors/bench-top-bioreactors/labfors-5
Econo-Pac® Chromatography Columns (Empty)	Bio-Rad	Cat#7321010
IndraDyn A Servomotor	Bosch Rexroth AG	SERVOMOTOR MAD100B-0250-SA-S2-AH0-05-A1 R911325822 https://store.boschrexroth.com/en/de/p/servomotor-r911325822
Lineareinheit	A-Drive Technology GmbH	
Mercury iTC503 temperature controller (sensor type ESR900)	Oxford Instruments NanoScience	https://nanoscience.oxinst.com/accessories/mercuryitc
Millex PVDF syringe filter	Merck Millipore	https://www.sigmaaldrich.com/DE/en/product/mm/slhv033n
Milli-Q IQ 7003 ultrapure and pure water purification system (or equivalent)	Merck Millipore	Cat#ZIQ7003T0C
Nanodrop 2000 Spectrophotometer	Thermo Fisher Scientific	Cat#ND-2000C
KORASILON® high viscosity silicone paste for sealing microcapillaries	Kurt Obermeier GmbH & Co. KG (vendor: Carl Roth GmbH + Co. KG)	Cat#0857.1
JEIO Tech ISS-7100 Stackable Incubator Shaker	Medline Scientific	Cat#AAH238132K
Thin Wall Quartz Q-band EPR Sample Tube (3 mm o.d., 250 mm length)	Wilmad LabGlass	Cat#705-SQ-250M
Thin Wall Quartz X-band EPR Sample Tube (4 mm o.d., 250 mm length)	Wilmad LabGlass	Cat#707-SQ-250M
Vibra cell sonicator	Sonics & Materials	Cat#VCX750
XDS-10 Vacuum Pump	Edwards	Cat#A73601983
Steinel hot air gun with LCD HG 2320 E	Carl Roth	Cat#ALN9.1

## Materials and equipment

**Timing: ∼ 2 h**

***Note:*** Unless otherwise stated, make fresh buffers for each sample preparation.

**Table:** Lysis buffer

Reagent	Final concentration/mM	Volume/mL
Na_2_HPO_4_ (0.5 M)	7	7
NaH_2_PO_4_ (0.5 M)	3	3
NaCl (0.5 M)	150	150
DTT (1 M)	10	5
Protease inhibitor cocktail (EDTA-free)	-	-
MilliQ H_2_O	-	335
**Total**		**500**

***Note:*** Add the protease inhibitor tablet as the last step. Perform all steps on ice. Store at 4°C for a maximum of 24 h.

**Table:** Refolding buffer

Reagent	Final concentration/mM	Volume/mL
Na_2_HPO_4_ (0.5 M)	7	7
NaH_2_PO_4_ (0.5 M)	3	3
NaCl (0.5 M)	150	150
L-arginine (1 M)	500	250
EDTA (0.1 M)	10	50
MilliQ H_2_O	-	40
**Total**		**500**

***Note:*** Store at 4°C for a maximum of 24 h.

**Table:** Denaturing buffer

Reagent	Final concentration/mM	Volume/μL
Na_2_HPO_4_ (0.5 M)	7	70
NaH_2_PO_4_ (0.5 M)	3	30
NaCl (0.5 M)	150	1500
Guanidine (12 M)	6000	2500
MilliQ H_2_O	-	900
**Total**		**5000**

***Note:*** Store at 4°C for a maximum of 24 h.

## Step-by-step method details

### MHQ sample preparation for MloK1 CNBD

**Timing: ∼ 2 h**

The following section describes MHQ sample preparation for the MloK1 CNBD and cAMP model system but can be extended to other protein-ligand pairs. It addresses: (i) the handling of both components for optimal ease-of-use during the MHQ sample acquisition, (ii) preparation and operation of the MHQ device, and (iii) packing and annealing of the sample in the EPR tube. At the end of this section, the sample is ready to be measured by PELDOR.

1. Centrifuge approximately 200 μL of CNBD solution (300 μM stock) and 500 μL of cAMP (30 mM stock) at 13,000 × *g* for 5 min at 4°C. Store samples on ice until use.

***Optional:*** To reduce aggregation and improve the deuteration efficiency of the sample matrix, add deuterated ethylene glycol (20% (v/v)) to both protein and ligand solutions.

***Note:*** This prolongs the electron-spin phase-memory time *T*_M_ and facilitates access to longer dipolar evolution times and, therefore, enables measurement of longer distances in PELDOR.^22^ However, note that cryoprotectants can alter structural equilibria, affect populations of conformational species,^3^^,^^23^^,^^24^ and increase the viscosity of the sample, potentially raising HPLC pump pressures. Always test cryoprotectant effects on the biomolecule’s stability and mixing efficiency.

2. Attach 100 μL sample loops to the MHQ device. Each MHQ time point requires 200 μL of CNBD solution (300 μM protein concentration) and 500 μL of cAMP (30 mM ligand concentration).

***Note:*** MHQ samples of the CNBD/cAMP system are prepared at a considerably lower volume than for the aging time calibration with MetMb/NaN_3_. This is because the subsequent PELDOR experiments will be performed at Q-band, which has an intrinsically higher concentration sensitivity than X-band.

***Optional:*** When connecting the sample loops, use a wrench to tighten (i.e., beyond finger-tight), as sample loops often eject under the high pressure formed upon switching the injection valve to the “inject” position.

**CRITICAL:** Ensure that there are no leaks after replacing the sample loops. Perform several mock injections of PBS buffer into the protein and the ligand channel and switch the injection valve position from “load” to “inject”. Robust connections should tolerate pressures of over 2000 psi without leakage.

3. Equilibrate the MHQ device in buffer (for details, see step 22 of the section “before you begin”).
4. Switch on the MHQ device (for details, see step 24 of the section “before you begin).
5. Start the software for controlling the cold-plate motor and the mixer-arm.
  a. Adjust the distance between the mixer and the cold-plate (for details, see steps 26a-c of the section “before you begin”).
  b. Check that the cold-plate can rotate freely (for details, see step 26e of the section “before you begin”).
6. Enter a program code into the LinControl software with an optimized mixer-arm wait-time. Refer to steps 26d and 27 of the section “before you begin” for details.

**CRITICAL:** Wear PPE (safety goggles or a face shield) for the next steps.

7. Pre-cool isopentane to a temperature of ≤ −130°C in a flat-bottom metal Dewar flask equipped with a magnetic stirring rod to prevent freezing (see section “before you begin”, step 35).

***Note:*** Use a thermometer to monitor the temperature during the following steps.

**CRITICAL:** The temperature of the isopentane bath must always remain ≤ −130°C to prevent sample thawing.

8. Pre-cool the funnel for sample packing (see step 36 of the section “before you begin”) and the cold-plate (see step 37 of the section “before you begin”).
9. Purge the protein and ligand Hamilton syringes, first with MilliQ H_2_O, followed by deuterated 1 × PBS buffer (see step 9a of the section “before you begin”).
  a. Draw 100 μL of the CNBD (300 μM) and the cAMP ligand (30 mM) into the syringes, taking care to remove air bubbles.
  b. Filter the samples before drawing them into the syringes to remove precipitates or other particulate matter that might clog the orifice.
    ***Alternatives:*** Separate any particulate matter by centrifugation (13,000 × *g*, 5 min, 4°C).
  c. Insert the syringes into the MHQ injection valves (in the “load” position).
10. Acquire the MHQ sample (see step 41 of the section “before you begin”, or Table 6).
11. Collect and pack the MHQ sample.
  a. Scrape the sample off the cold-plate walls (see step 41g of the section “before you begin”) and transfer the sample into the funnel (see step 42 and 45 of the section “before you begin”).
  b. Pack the sample in a 3 mm o.d. EPR tube (see step 45 of the section “before you begin”).
12. Anneal the sample tube as described in step 53b of the section “before you begin” to remove N_2__(l)_ trapped in sample voids. Compress the sample with a packing rod, cryogenically cooled in N_2__(l)_. Troubleshooting 2**.**

### Acquisition and analysis of MHQ/PELDOR data

**Timing: ∼3 days**

Pulse dipolar EPR spectroscopy (PDS) is a technique to measure the distance between unpaired electrons in the range of 1.5–16 nm with Angstrom precision.^22^^,^^25^^,^^26^^,^^27^ The PDS toolbox encompasses several experiments,^28^ the most popular being PELDOR. PELDOR is typically performed on ensembles of biomolecules in frozen solution and is exclusively sensitive to unpaired electron spins, i.e., it is blind to the diamagnetic “background”. The long-range distance constraints provided by PELDOR are complementary to the atomic resolution achieved by X-ray crystallography and cryo-EM, but without necessitating crystallization or surface immobilization. Furthermore, PELDOR is virtually unrestricted with respect to the size of the biomolecule, facilitating the study of complex molecular machinery^29^^,^^30^^,^^31^ with the ribosome^32^ being the largest molecular complex studied by PELDOR, to date. Of note, the nitroxide spin labels most often used for PELDOR are comparatively small and structurally less perturbing than the sterically bulkier fluorophores used in FRET.^33^ In this purview, PELDOR has been extensively applied to proteins and nucleic acids, characterizing (i) conformational dynamics,^34^^,^^35^^,^^36^ (ii) domain rearrangements,^37^ (iii) binding equilibria,^38^^,^^39^^,^^40^^,^^41^ (iv) oligomerization,^42^^,^^43^ and (v) the kinetics of these processes.^1^^,^^44^^,^^45^^,^^46^

PELDOR/DEER measures the dipolar-coupling frequency ν_dip_ between the spins of unpaired electrons,^22^ which is inversely proportional to the cube of the inter-spin distance r (Equation 19):

(Equation 19) $$\nu_{Dip}=\frac{\mu_{0}}{4\pi h}\cdot \frac{1}{r^{3}}\cdot g_{1}g_{2}\beta_{e}^{2}\left(1-3\;\cos^{2}\theta \right)$$

Herein, μ_0_ is the vacuum magnetic permeability, h the Planck constant, g_1_ and g_2_ the g-factors of the two dipolar-coupled unpaired electron spins (g_1_ **∼** g_2_ **∼** g_e_), β_e_ is the Bohr magneton, and θ the angle between the vector connecting the two spins and the direction of the external magnetic field.

The primary time-domain data, so-called dipolar traces that encode the dipolar coupling, can be transformed into distance probability-density distributions P(r) that report on the conformational ensemble of the biomolecule. PELDOR is well-suited especially for identifying sub-ensembles within a conformational landscape and for monitoring structural changes that are coupled to external stimuli such as pH,^47^^,^^48^ ligand-binding,^49^^,^^50^^,^^51^^,^^52^ ionic strength,^53^^,^^54^ and the environment of the biomolecule such as native membranes or membrane-mimetic environments^55^^,^^56^^,^^57^^,^^58^^,^^59^^,^^60^ and entire cells.^61^^,^^62^^,^^63^^,^^64^ In favorable cases, PELDOR allows quantification of conformational sub-populations in an ensemble, e.g., by integrating peaks in the distance probability-density distribution P(r).^65^^,^^66^^,^^67^ However, such analysis is confounded by regularization procedures to transform the experimental dipolar trace into the distance domain. It is complicated further if the distribution peaks are not well-separated (i.e., unclear start- and end-points for integration). Instead, direct quantification from background-corrected time-domain data may be preferable^1^^,^^68^^,^^69^ under conditions where (i) the conformational change is coupled to an external trigger such as a binding event (i.e., experimental traces are deconvoluted into a linear combination of *apo*- and *holo*-states), and (ii) the contributions of intermolecular and intramolecular dipolar coupling to the trace can be isolated robustly.^70^

Herein, we describe a combination of MHQ and PELDOR (MHQ/PELDOR) to track cAMP-induced conformational changes of a CNBD construct containing a pair of exogenously introduced cysteine residues (E289C/I340C) over space and time. We analyze MHQ/PELDOR data with the deconvolution approach using a home-written graphical user interface (GUI) with automated report generation to determine the *apo*- and *holo*-state populations from experimental dipolar traces. The discussion will focus on aspects that distinguish the MHQ/PELDOR set-up, sample handling, and data acquisition from conventional PELDOR measurements on nitroxide-labeled biomolecules. The following section summarizes the set-up of the four-pulse PELDOR experiment at Q-band (∼34 GHz) on a commercial ELEXSYS E580 EPR spectrometer (Bruker BioSpin, Ettlingen, Germany) equipped with a Flexline probehead and an ER5106 QT-II resonator, a 150 W traveling wave tube (TWT) amplifier (model: 187Ka, Applied Systems Engineering, Fort Worth, Texas, USA), and the Xepr software (Bruker). For detailed step-by-step instructions, the reader is referred to several excellent protocols^71^^,^^72^^,^^73^^,^^74^^,^^75^ and the instrument manual.

13. Cool down the cryostat of the spectrometer to a temperature of 50 K using liquid helium. For methodological details, refer to step 53c of the section “before you begin”.
14. Mount the sample tube into the sample holder, positioning the center of the sample at 38 mm from the bottom of the holder.

***Note:*** This ensures the active volume of the resonator is filled with sample (i.e., where the B_1_ field is homogeneous).

**CRITICAL:** Do not submerge the entire tube, as N_2(l)_ will again permeate within sample voids, otherwise repeat the annealing procedure. Also, note that the positioning of the sample center depends on the resonator; consult the user manual.

15. Quickly (maximally 5-10 s) take the sample tube out of the Dewar flask, carefully shake it to remove residual nitrogen from the tube surface, and mount it into the spectrometer.

***Note:*** To minimize the transfer path from the Dewar flask to the spectrometer and thus the time the sample tube is exposed to ambient temperature, one can position the Dewar flask between the magnet coils.

**CRITICAL:** Set the magnetic field of the spectrometer to 0 G before moving between the solenoid coils, as Dewar flasks are magnetic.

***Note:*** Throughout the following steps, the names of windows and functions in the Xepr software will be indicated by quotation marks (“, ”).

16. Over-couple the resonator as described in the user manual.
  a. Perform the safety check to ensure that protection gates prevent the detector from the high-power microwave pulses.
  b. If the protection gates are present, switch the traveling wave tube (TWT) amplifier into the “Operate” mode.
17. Perform set-up experiments.
  a. Set the “Center Field” (“Field” tab in the “FT EPR Parameters” window) to the value that corresponds to g ∼ 2.0 at the given microwave frequency (e.g., 11980 G at 33.7 GHz).
  b. Record the echo-detected field swept (EDFS) EPR spectrum.
    i. Program the sequence (*π*/2 −*τ* −*π* −*τ* −*echo*) into the “+x” channel of the Pulse Tables (“Patterns”) using 12 ns, 24 ns, and 200 ns for the π/2-pulse, the π-pulse, and *τ*, respectively.
    ii. In the “Acquisition Trigger” channel, set the “Position” to 0 ns and the “Length” to 4 ns.
    iii. Set the “Integrator Time Base” to 1.0 ns, the number of “Shots Per Point” to 10, and the shot-repetition time (“Shot. Rep. Time”, SRT) to 2000 μs.
      ***Note:*** An SRT of 2000 μs is typically a good starting point for pulsed EPR experiments on nitroxides at 50 K and Q-band frequency.
    iv. Set the “Video Bandwidth” to 20 MHz, click the “Start” button in the Pulse Tables window and the “Run” button in “SpecJet”, and decrease the microwave “Attenuation”.
      ***Note:*** If the echo amplitude has not gone through a maximum even upon lowering the microwave attenuation to 0 dB, increase the pulse lengths (e.g., to 16 ns and 32 ns for the π/2- and π-pulse, respectively) and repeat step 17b. Also, check the tuning of the resonator.
      **CRITICAL:** The SRT must be long enough to yield at least 80% of the echo amplitude obtained after complete longitudinal spin-relaxation; this prevents saturation of the spin system. Test several SRT values between ∼1000 μs and ∼5000 μs and check the echo amplitude in the “SpecJet” oscilloscope.
    v. Monitor the Hahn echo in “SpecJet” and adjust the microwave “Attenuation” to maximize the echo amplitude, i.e., to obtain π/2- and π-pulses at the given pulse lengths.
      ***Note:*** Normally, ∼3 dB microwave attenuation is appropriate to obtain π/2- and π-pulses of 12 ns and 24 ns.
    vi. Adjust the “Center Field” by up to ±10 G to bring the sample fully on resonance (i.e., to maximize the Hahn echo amplitude).
    vii. Optimize the “Signal Phase” so that the in-phase component of the signal (real channel, green trace in “SpecJet”) is maximized.
      ***Note:*** The amplitude in the imaginary channel (yellow trace in “SpecJet”) should be brought close to zero by optimizing the “Center Field” and the “Signal Phase”.
    viii. Set the number of averages to unity in “SpecJet”. Optimize the “Video Gain” amplification such that the signal is maximized but not clipped (i.e., no signal truncation in “SpecJet”).
    ix. In the “Acquisition” tab of the “FT EPR Parameters” window, select “X-Axis Quantity: Magnetic Field”.
    x. Integrate the entire echo by setting the “Acquisition Trigger” “Position” to approx. 790 ns with a “Length” of approx. 120 ns (the timing of the acquisition trigger depends on the spectrometer dead time and might vary).
      **CRITICAL:** Integrating the entire echo is crucial when recording the EDFS EPR spectrum to obtain a sufficient field resolution.^76^
    xi. Set the “Center Field” to the value that yields the maximal echo intensity and set the “Sweep Width” to 400 G.
    xii. Record two points per G (i.e., 800 points in this example) on the abscissa (to be entered into the “X-Axis Size” window in “FT EPR Parameters”).
    xiii. Press the “Run” button in the main window to start the acquisition and save the EDFS on disk.
      ***Note:*** Depending on the spin concentration, it might be necessary to accumulate some scans (typically 3-5) to obtain a sufficient signal-to-noise ratio (SNR).
      ***Optional:*** To fully optimize the SRT, one can run an inversion-recovery experiment (see step 17c).
  c. Perform an inversion-recovery experiment (*π*_*inv*_ −*T* −*π*/2 −*τ* −*π* −*τ* −*echo*) to determine the longitudinal electron-spin relaxation time (*T*_1_).
    ***Note:*** In the inversion-recovery experiment, the interval *T* is incremented, and the echo amplitude is monitored as a function of *T*.
    i. Set the “Center Field” to the magnetic field value of the signal maximum in the EDFS EPR spectrum.
    ii. Open the PulseSPEL editor by clicking the “PulseSPEL” button in the “FT EPR Parameters” window.
    iii. Load the PulseSPEL program (“InvRec_ESE.exp”) and the variable definitions (“descrRelaxation.def”) for the inversion recovery experiment.
      ***Note:*** These files are shipped with the spectrometer and located by default in the directory “xeprFiles/PulseSPEL/sharedPulseSPEL/Standard/Relaxation”.
    iv. Perform the experiments given in the PulseSPEL program. First, update the variable definitions for the inversion-recovery setup experiments using the parameters in Table 8.
      ***Note:*** Herein, *p*0 is the length of the π/2-pulse, *p*1 is the length of the π-pulse, *p*2 is the length of the inversion pulse π_inv_, *d*0 is the instrument-dependent acquisition trigger delay, *d*1 is the interpulse delay *τ* between the π/2- and the π-pulse in the Hahn echo sub-sequence, *d*2 is the interpulse delay *T* between the inversion pulse π_inv_ and the π/2-pulse, *d*30 is the time increment of *p*2 or *d*2 (depending on the particular experiment), *h* is the number of shot-per-point, *n* is the number of scans to accumulate, and *SRT* is the shot-repetition time.
    v. Select the “2P ESE Setup” PulseSPEL program (*π*/2 −*τ* −*π* −*τ* −*echo*) from the drop-down list and click the buttons “Compile”, “Show Program”, and “Validate” in the given order.
    vi. Press the “Run” button in the Xepr main window. Inspect the Hahn echo in the SpecJet oscilloscope and maximize the in-phase component (real channel, green trace) by slightly changing the microwave phase.
      ***Note:*** The signal in the imaginary channel (yellow trace) should be minimized.
    vii. Read off when the echo maximum occurs, add this value to *d*0, and update *d*0. Press the “Run” button in the Xepr main window again. Set the acquisition trigger to the echo maximum.
      ***Note:*** The first point of the echo displayed in the Xepr viewport should have the highest signal intensity. If this is not the case, adjust *d*0 accordingly.
    viii. Select the “Inversion Recovery Setup” program (*π*_*inv*_ −*T* −*π*/2 −*τ* −*π* −*τ* −*echo*) and press the “Run” button in the Xepr main window. Set the acquisition trigger to the echo maximum.
      ***Note:*** The displayed echo will be inverted due to the inversion pulse applied before the Hahn-echo sequence (inverted Hahn echo).
    ix. Select the “Inversion Pulse Optimization” program (*π*_*inv*_ −*T* −*π*/2 −*τ* −*π* −*τ* −*echo*) and press the “Run” button in the Xepr main window.
      ***Note:*** In this experiment, the length of the inversion pulse *π*_*inv*_ is incremented and the echo amplitude is recorded as a function of *π*_*inv*_ (nutation experiment). *π*_*inv*_ should invert the magnetization maximally (typically, *π*_*inv*_ ∼ 24 ns)
    x. Read off the length of *π*_*inv*_ that gives the first minimum in the nutation trace. Set this value for *p*2 in the PulseSPEL program.
    xi. Select the “Inversion Recovery” program (*π*_*inv*_ −*T* −*π*/2 −*τ* −*π* −*τ* −*echo*). Set the parameters given in Table 9:
      ***Note:*** In the inversion-recovery experiment, the interpulse delay *T* is incremented, and the echo amplitude is recorded as a function of *T*.
    xii. Set the number of points to be recorded on the abscissa (parameter dim4 in the PulseSPEL program) to 820 and click the buttons “Compile”, “Show Program”, and “Validate” in the given order. Press the “Run” button in the Xepr main window to record the inversion-recovery trace.
      ***Note:*** The inversion-recovery trace plateaus at long interpulse delays *T*. This time *T* corresponds to the time it takes the spin system to recover to the equilibrium magnetization.
    xiii. Determine the signal intensity of the plateau. Calculate 80% of the plateau amplitude and read off the associated time. Set this value for the SRT for subsequent experiments.
18. Perform the two-pulse electron-spin echo envelope modulation (two-pulse ESEEM) experiment to determine the electron-spin phase-memory time (*T*_M_).
  ***Note:*** In the two-pulse ESEEM experiment, the amplitude of the Hahn echo is recorded as a function of the interpulse delay *τ*. The rate of the echo decay upon incrementing τ provides information on transverse electron-spin relaxation, which limits the length of the dipolar trace in the subsequent PELDOR experiment.
  a. Set the “Center Field” to the magnetic field value of the signal maximum in the EDFS EPR spectrum.
  b. Open the PulseSPEL editor by clicking the “PulseSPEL” button in the “FT EPR Parameters” window. Load the PulseSPEL program (“2p_ESEEM.exp”) and the variable definitions (“descrESEEM.def”) for the two-pulse ESEEM experiment.
    ***Note:*** These files are shipped with the spectrometer and located by default in the directory “xeprFiles/PulseSPEL/sharedPulseSPEL/Standard/ESEEM”. The default PulseSPEL program delivered with the spectrometer records the Hahn echo amplitude as a function of the interpulse delay *τ*. However, by convention, it should be analyzed and published as a function of 2*τ*.^22^^,^^77^
  c. Update the two-pulse ESEEM variable definitions using the parameters given in Table 10.
    ***Note:*** Herein, *p*0 is the length of the π/2-pulse, *p*1 is the length of the π-pulse, *d*0 is the instrument-dependent acquisition trigger delay, *d*1 is the initial interpulse delay *τ* in the Hahn echo sequence, *d*30 is the time increment of *d*1, *h* is the number of shot-per-point, *n* is the number of scans to accumulate, and SRT is the shot-repetition time.
  d. Compile the parameters.
    i. In the PulseSPEL editor, click the buttons “Compile”, “Show Program”, and “Validate” in the given order.
    ii. In the “Acquisition” tab of the “FT EPR Parameters” window, choose “Acquisition Mode: Run from PulseSPEL” and select “2P ESE Setup” from the list of experiments.
  e. Record the Hahn echo by clicking the “Run” button in the Xepr main window.
    i. Check that the acquisition trigger is set to the echo maximum.
    ii. Monitor the echo in the SpecJet oscilloscope and optimize the microwave phase so that the in-phase component (real channel, green trace in SpecJet) is maximal.
  f. Set the number of averages to one in “SpecJet”. Adjust the “Video Gain” amplification so that the signal is maximal but not clipped (i.e., no signal truncation in SpecJet).
  g. Record the two-pulse ESEEM trace.
    i. Set the number of points on the abscissa by adjusting the value of “dim2” in the PulseSPEL program (e.g., dim2 = 1024 points at a time-step of 8 ns yields a trace length of ∼8 μs).
      ***Note:*** The trace should be long enough to monitor a decay to ∼1% of the initial echo amplitude; increase *dim*2 and *d*30 correspondingly, if necessary.
    ii. In the “Acquisition” tab of the “FT EPR Parameters” window, select the “2P ESEEM” experiment from the drop-down menu and a “Two Step” phase cycle.
    iii. Execute the experiment by clicking “Run” in the Xepr main window.
      ***Note:*** Changing the number of scans (PulseSPEL parameter *n*) is still possible during acquisition using the “PulseSPEL Variable” box in the “Acquisition” tab of the “FT EPR Parameters” window.
    iv. Determine the length of *τ* when the echo has decayed to ∼5% of its initial intensity. Determination of this time by visual inspection is usually sufficient.
      ***Note:*** Write down this value. It is a good starting point for setting the dipolar evolution time in the subsequent PELDOR experiment, and it roughly equals the (1/(2e^2^)) time.
    v. Save the two-pulse ESEEM trace on disk. If the SNR is insufficient, increase the number of scans and restart the experiment.
19. Perform the PELDOR experiment. Troubleshooting 3**.**
  ***Note:*** PELDOR is a pump-probe EPR experiment that involves two microwave frequencies, the pump frequency ν_pump_ and the probe frequency ν_probe_. In a system of two coupled spins A and B, a refocused Hahn echo (RE) is recorded on spin A via the probe sequence π/2–τ_1_–π–(τ_1_ + τ_2_)–π–τ_2_–RE, which is applied at ν_probe_ (see Figures 4G and 4H). Within the first interval τ_2_, spin B is inverted by a π-pulse at ν_pump_. Incrementing the position of the pump pulse within τ_2_ yields a modulation of the RE, with the frequency of this modulation encoding the dipolar coupling between spins A and B. At Q-band and for nitroxides, the difference between ν_pump_ and ν_probe_ is usually set to ∼80 to ∼100 MHz (see Figure 4H).
  a. Load the program and the variable definitions for the PELDOR experiment into PulseSPEL and click the buttons “Compile”, “Show Program”, and “Validate”.
  b. Set the following pulse lengths and interpulse delays in PulseSPEL:
    i. For the π/2- and π-pulse of the probe sequence (variables *p*0 and *p*1 in PulseSPEL), set the values proven optimal for the Hahn echo at step 17.
    ii. Set *τ*_1_, (PulseSPEL variable *d*1) to the value that yields the global maximum in the two-pulse ESEEM trace.
      ***Note:*** For the interpulse delay *τ*_1_, values between 200 ns and 400 ns have been reported.^78^^,^^79^
  c. In the “Acquisition” tab of the “FT EPR Parameters” window, select “Experiment: 2P ESE Setup”, which is a PulseSPEL program for the Hahn echo.
    i. Set the “Attenuation” to 0 dB. Click “Run” in the Xepr main window, “Start” in the Pulse Tables window, and “Run” in SpectJet.
    ii. Go to the “MPFU Control” tab of the “FT Bridge” window and increase the “<+x> Amplitude” to maximize the Hahn echo displayed in SpecJet.
      ***Note:*** For our instrument, at π/2 = 12 ns, the <+x> Amplitude is typically at ∼68% when the Hahn echo is maximized.
  d. Adjust the “Signal Phase” (“Receiver Unit” tab in the “FT Bridge” window) so that the signal is maximized in the real channel (green trace in SpecJet).
20. Optimize the ELDOR frequency and attenuation.
  a. In the “Microwave” tab of the “FT EPR Parameters” window, set the “Current ELDOR Freq.” to the current microwave frequency (∼33.7 GHz) and write down this value.
    ***Note:*** This frequency corresponds to ν_pump_ in the PELDOR experiment. In this experiment, the pump pulse (variable *p*2 in the PulseSPEL program) is applied at ν_pump_ before the Hahn echo sequence, which is performed at ν_probe_.
  b. Select the “3P ELDOR Setup” experiment from the drop-down menu.
    i. Click the “Run” button in the Xepr main window and observe the Hahn echo in SpecJet.
    ii. Stepwise decrease the “ELDOR Attenuation” to 0 dB (“Microwave” tab in the “FT EPR Parameters” window).
    iii. Adjust *d*0 so that the acquisition trigger is at the echo maximum (typically, *d*0 = 432 ns).
21. Run a transient nutation experiment.
  ***Note:*** This experiment records the Hahn echo amplitude as a function of the pump-pulse length (π_pump_, PulseSPEL variable *p*2). Determining the pump-pulse length that leads to maximal inversion of the echo yields optimal modulation depth and PELDOR sensitivity.
  a. Select the “3P ELDOR Nutation” experiment from the drop-down menu and press the “Run” button in the Xepr main window to record the nutation trace.
  b. Save the trace on the disk and read off the pulse length that maximally inverts the echo (typically between 12 ns and 18 ns).
  c. Set this value for the pump-pulse length in the PELDOR experiment (PulseSPEL variable *p*2).
    ***Note:*** If the spectrometer is not equipped with an ELDOR unit, the transient nutation and the PELDOR experiments can also be run using an Arbitrary Waveform Generator (AWG).
    **CRITICAL:** Careful determination of π_pump_ is crucial for the subsequent PELDOR experiment. Inappropriate values of π_pump_ will reduce the modulation depth and worsen the SNR.
22. Change the spectrometer frequency from ν_pump_ to ν_probe_.
  a. Set the “Attenuation” to 60 dB and the “ELDOR Attenuation” to 30 dB.
  b. Switch the TWT amplifier into “Standby” mode. Wait until the cathode voltage has dropped to 0 V before you proceed.
  c. In the “Bridge Configuration” tab of the “FT Bridge” window, switch the microwave bridge to continuous-wave (“CW”) mode.
  d. Open the “Microwave Bridge Tuning” panel and lower the current “Frequency” of the spectrometer by the desired offset Δ*ν*.

***Note:*** The offset Δ*ν* should be neither too small (i.e., to prevent overlap of the excitation profiles of the pump and probe pulses) nor too large (i.e., to obtain a sufficiently intense signal). Setting Δ*ν* to −80 or −100 MHz is typically appropriate at Q-band.

23. Optimize the Hahn echo at the probe frequency ν_probe_.
  a. Switch back to pulse mode (“Bridge Configuration” tab of the “FT Bridge” window) and fine-adjust the probe frequency to match the desired offset.
  b. Activate the FCL option (“Options” button in the “Microwave Bridge Tuning” panel) to stabilize the microwave frequency.
  c. Perform the safety check as described in the spectrometer manual and switch the TWT amplifier to “Operate” mode.
  d. Set the acquisition trigger delay (variable *d*0 in PulseSPEL) to 360 ns.
  e. From the drop-down menu (“Acquisition” tab in the “FT EPR Parameters” window), select “Experiment: 2P ESE Setup” and the phase cycling option “+<x> none”.
  f. Press the “Run” button in the Xepr main window, “Start” in the Pulse Tables window, and “Run” in “SpecJet”.
  g. Decrease both the “Attenuation” and the “ELDOR attenuation” stepwise to 0 dB and adjust the “Video Gain” to prevent clipping of the signal.
  h. Fine-adjust the “+<x> Amplitude” slider bar in the “MPFU Control” tab of the “FT Bridge” window to maximize the echo amplitude (typically ∼72% of the slider bar) in the real channel (green trace in SpecJet).
    ***Note:*** Adjust the “+<x> Phase” slider bar so that the real part of the signal is negative-going.
  i. Select the “–<x> none” phase cycling option and press “Run” in the Xepr main window. Adjust the “–<x> Amplitude” slider bar to maximize the echo (typically ∼72% of the slider bar) in the real channel (green trace in SpecJet).
    ***Note:*** Adjust the “–<x> Phase” slider bar so that the real part of the signal is positive-going.
    ***Note:*** For both phase cycling options, the imaginary part of the signal (yellow trace in SpecJet) should be zero on average. Also, check that the echoes of the phase cycling options “+<x> none” and “–<x> none” have comparable absolute amplitudes.
24. Decide on the dipolar evolution time *τ*_2_ (PulseSPEL variable *d*2) for the PELDOR experiment and optimize the refocused echo.
  a. Choose a dipolar evolution time *τ*_2_ (i.e., the length of the PELDOR trace; see Figure 4F) that is appropriate for the specific sample and set it as the PulseSPEL variable *d*2.
    ***Note:*** As a rule of thumb, set a dipolar evolution time that resolves ≥1.5 periods of the expected dipolar oscillation.^22^ The period of one dipolar oscillation, *t*_*Dip*_, can be calculated by inverting the fraction in Equation 19. This leads to Equation 20:
    (Equation 20) $$t_{Dip}=\frac{1}{\nu_{Dip}}=\frac{4\pi h}{\mu_{0}g_{1}g_{2}\beta_{e}^{2}\left(1-3\;\cos^{2}\theta \right)}\cdot r^{3}$$
    Setting *θ* = 90° yields Equation 21:
    (Equation 21) $$t_{Dip}=1.926\cdot 10^{19}\frac{s}{m^{3}}\cdot r_{\max}^{3}=1.926\cdot 10^{-5}\frac{\mu s}{{Å}^{3}}\cdot r_{\max}^{3}$$
    where *r*_*max*_ is the maximal distance to be resolved.^22^ Thus, to resolve ≥ 1.5 oscillation periods, the following inequality must be fulfilled:
    (Equation 22) $$t\geq 2.889\cdot 10^{-5}\frac{\mu s}{{Å}^{3}}\cdot r_{\max}^{3}$$
    If the distance distribution is unknown *a priori*, use the value determined at step 18 for the dipolar evolution time and run a preliminary PELDOR experiment. If a longer dipolar trace is needed to resolve 1.5 oscillations or if the time window is too long and the SNR concomitantly low, adjust the dipolar evolution time accordingly. In general, the experimentally accessible dipolar evolution time is limited by transverse electron-spin relaxation, and the minimal dipolar evolution time required for data analysis is dictated by the expected interspin distance.^22^ A longer dipolar evolution time resolves more dipolar oscillations, eases background fitting, and thus provides more reliable primary data. This, however, comes at the expense of increased acquisition times or a lower SNR.

**Table 11:** Parameters for the PELDOR experiment on nitroxides at 50 K at Q-band

Parameter	PulseSPEL variable	Explanation/Value
π/2_probe_	p0	12 ns
π_probe_	p1	24 ns
π_pump_	p2	determined at step 21
-	pg	Integrator gate width; length of the longest pulse in sequence
-	d0	Acquisition trigger offset; determined at step 24g
τ_1_	d1	Between 200 ns and 400 ns; e.g., the time value of the global maximum in the two-pulse ESEEM trace, 232 ns
τ_2_	d2	determined at step 18g, sub-step iv
-	d3	Spectrometer dead time, 100 ns
-	d30	Increment of the dipolar evolution time; determined at step 25b
-	d31	Increment for nuclear modulation averaging, 16 ns to suppress ^2^H ESEEM at Q-band
-	SRT	Shot-repetition time, 2000 ∗ srtu
-	h	Number of shots-per-point, 10
-	n	Number of averages, as required for sufficient SNR
-	m	Number of nuclear modulation averaging steps, 8 to suppress ^2^H ESEEM at Q-band
-	PC	2-step phase-cycling
-	dim5	Number of points on the abscissa, determined from Equation 23
  b. Select the “4P DEER Setup” experiment, the phase cycling option “2-step”, and press the “Run” button in the Xepr main window.
  c. Click “Start” in the Pulse Tables, “Run” in SpecJet, and observe the refocused echo (RE, see Figure 4G).
  d. Set the number of transient averages in SpecJet to one and adjust the Video Gain amplification so that the echo is not clipped. Increase the number of averages in “SpecJet” to obtain a higher SNR (100-1000 averages) and click “Run” in the Xepr main window.
  e. Read off the time of the echo maximum and write it down.
  f. Set the integration gate width (PulseSPEL variable *pg*) to the length of the longest pulse in the PELDOR sequence (e.g., 24 ns of the π_probe_-pulse) to maximize the SNR.^76^
  g. Adjust the acquisition trigger offset (PulseSPEL variable *d*0) so that the integration gate is centered symmetrically around the echo maximum.
25. Select the “4P DEER” experiment to record the PELDOR trace and set the acquisition parameters described in the following sub-steps. An overview of all parameters is given in Table 11.
  a. Set the number of steps for nuclear modulation averaging (PulseSPEL variable *m*) and the corresponding time increment (PulseSPEL variable *d*31).
    ***Note:*** At Q-band, *m* = 8 and *d*31 = 16 ns are appropriate if the buffer and/or the protein are deuterated.
    ***Note:*** Nuclear modulation averaging is required to suppress deuterium ESEEM in the PELDOR trace. Averaging 8 steps over one ESEEM oscillation period usually leads to almost complete suppression of ESEEM. The time increment for modulation averaging can be computed by (1/*ν*_*Larmor*_)/*m*, where *ν*_*Larmor*_ is the nuclear Larmor frequency of deuterium at the given magnetic field.
  b. Decide on the time resolution of the PELDOR trace (PulseSPEL variable *d*30) taking the Nyquist-Shannon sampling theorem into account.^22^^,^^80^^,^^81^
    ***Note:*** Generally, the faster the dipolar oscillation is, the shorter *d*30 must be to reliably resolve the oscillation (i.e., to mitigate aliasing artifacts). However, setting *d*30 excessively short combined with a long dipolar evolution time will lead to (unnecessarily) long acquisition times. Therefore, decide on *d*30 based on the oscillation period and the dipolar evolution time.
  c. Set the number of scans (variable *n* in PulseSPEL) sufficiently high (e.g., *n*=10,000, depending on the sample and the chosen acquisition parameters).
    ***Note:****n* can still be changed while the experiment is running.
  d. Determine the number of points to be recorded on the abscissa using Equation 23 and set this value for dim5 in the PulseSPEL program:
    (Equation 23) $$\dim\;5=\frac{d1+d2-2d3}{d30}$$
    ***Note:****d*1 is the interpulse delay *τ*_1_, *d*2 is the dipolar evolution time *τ*_2_, *d*3 is the spectrometer dead time delay, and *d*30 is the time-increment of the dipolar evolution time. Table 11 summarizes the parameters required for the PELDOR experiment at Q-band.
  e. Run the “4P DEER” experiment.
    i. Select the “2-step” phase cycling option, and click “Run” in the Xepr main window.
    ii. Press the “Re/Im” button in the Xepr main window to display the imaginary part of the dataset in the viewport.
      ***Note:*** The imaginary part should fluctuate around the zero level if the MW phase has been adjusted properly; otherwise, slightly change the “Signal Phase” in the “Receiver Unit” tab of the “FT Bridge” window, abort the experiment, and restart it.
  f. Let the experiment acquire. Stop the experiment and save the dipolar trace to disk when the SNR is sufficiently high (preferably > 20). Troubleshooting 3**.**
    ***Note:*** The SNR of the dipolar trace will increase with the square root of the acquisition time. Depending on the dipolar evolution time, transverse electron-spin relaxation, and spin concentration, the PELDOR experiment usually takes between two and 96 h. Troubleshooting 3.
    ***Note:*** For reliable data analysis, the SNR should exceed the threshold of 20.^22^ Here, the SNR is given by SNR = Δ/*σ*, with *σ* being the standard deviation of the noise determined from the imaginary part of the dataset and Δ being the modulation depth.^22^ The approximate duration of the experiment can be calculated using Equation 24:
    (Equation 24) Acquisitiontime=SRT·PC·m·h·n·dim5
26. Remove the sample from the cryostat.
  a. Drag all MPFU channel slider bars to zero (“MPFU Control” tab in the “FT Bridge” window).
  b. Set the “Attenuation” to 60 dB, the “ELDOR Attenuation” to 30 dB, and the TWT into Standby mode.
  c. Switch to “CW” mode (“Bridge Configuration” tab in the “FT Bridge” window), adjust the MW frequency to 33.7 GHz, and set the MW Bridge to “Standby” (“Microwave Bridge Tuning” panel).
  d. Switch off the membrane pump and wait for the negative pressure to return to ambient pressure.
  e. Remove the sample rod from the cryostat. Transfer the sample quickly into a Dewar flask filled with N_2(l)_.
27. Either repeat the protocol starting from step 7 or switch off the spectrometer as described in the user manual.
28. Process the acquired PELDOR data with the DeerAnalysis package.^21^

***Note:*** This is the most popular software for analyzing PELDOR data^82^ and was used in the original publication on MHQ/PELDOR.^1^

***Alternatives:*** Use the ComparativeDeerAnalyzer software (CDA).^83^^,^^84^^,^^85^ This tool provides a one-step conversion of the experimental dipolar trace into a distance distribution and performs an automatic background correction, thereby minimizing operator bias. One-step conversion also avoids noise explosion^86^ and is therefore particularly useful for MHQ samples, as dilution from condensation during sample acquisition^14^^,^^87^ can limit the SNR.

***Note:*** The individual scans of the PELDOR measurement can be saved automatically using a ProDEL script available on Github to avoid data loss in case of power outages, temperature instabilities, and software crashes.

**CRITICAL:** Use the background-corrected dipolar traces for the following steps.

29. Deconvolute the experimental dipolar trace recorded on the MHQ sample to assess the *apo*- and *holo*-state contributions by solving Equation 25:
  (Equation 25) $$\vec{MHQ}=a\cdot \vec{Apo}+b\cdot \vec{Holo}$$
  ***Note:***$\vec{MHQ}$, $\vec{Apo}$, and $\vec{Holo}$ are vectors providing the dipolar traces (signal intensity *vs.* discrete dipolar evolution time) of the MHQ sample at a defined aging time, the *apo*-state, and the *holo*-state, respectively. The scalars *a* and *b* denote the fractions by which the *apo-* and *holo*-trace must be mixed *in silico* to reproduce the MHQ trace.
  a. Open the deconvolver GUI, select toggle switches depending on the desired output (see **Note** below), and press “RUN”. Dialog boxes will open for selecting ASCII files containing the “*apo-*”, “*holo*-”, and “MHQ” dipolar traces.
    ***Note:*** Toggle switches within the GUI include: (i) “force a+b=1” yielding direct populations of *apo*-state and *holo*-state (if this switch is off, a reminder will appear to normalize the coefficients before interpretation), (ii) “save ASCII files”, (iii) “save PNG files”, and (iv) “generate report”. Options (ii) and (iii) save the original input traces, the modulation-depth scaled traces, the “mix trace” obtained *in silico* as a linear combination of the *apo-*state and the *holo-*state trace, and the residual in ASCII format and as 600 dpi resolution PNG figures, respectively. Option (iv) saves the figures, metadata, and coefficients into a PDF report.
  b. The *“holo*-state” and “MHQ” dipolar traces are scaled to the same modulation depth as the “*apo*-state” trace^21^^,^^88^ using Equation 26:
    (Equation 26) $$f^{\lambda }=\left\{ \begin{array}{c} \frac{\sum_{k=1}^{\left\lfloor \frac{\max\left(N-1\right)}{\max\left(\alpha \right)}\right\rfloor }{\left[\ln\;V_{1}\left(t_{\left(\max\left(\alpha \right)k\right)}\right)\right]}^{2}}{\sum_{k=1}^{\left\lfloor \frac{\max\left(N-1\right)}{\max\left(\alpha \right)}\right\rfloor }\ln\;V_{1}\left(t_{\left(\max\left(\alpha \right)k\right)}\right)\ln\;V_{2}\left(t_{\left(\min\left(\alpha \right)k\right)}\right)},\Delta t_{V_{2}}>\Delta t_{V_{1}}\\ \frac{\sum_{k=1}^{\left\lfloor \frac{\max\left(N-1\right)}{\max\left(\alpha \right)}\right\rfloor }{\left[\ln\;V_{1}\left(t_{\left(\min\left(\alpha \right)k\right)}\right)\right]}^{2}}{\sum_{k=1}^{\left\lfloor \frac{\max\left(N-1\right)}{\max\left(\alpha \right)}\right\rfloor }\ln\;V_{1}\left(t_{\left(\min\left(\alpha \right)k\right)}\right)\ln\;V_{2}\left(t_{\max\left(\alpha \right)k}\right)},\Delta t_{V_{1}}>\Delta t_{V_{2}} \end{array}\right.$$
    ***Note:*** Here, *f*^*λ*^ is the scaling factor, *Ν* is the number of data points, *k* indexes from the first non-zero time point, *V*_1_ is the “*apo*-state” dipolar trace, and *V*_2_ is the “*holo*-state” or “MHQ” dipolar trace.^21^^,^^88^ Here, *α* is the downsampling factor, defined by Equation 27:
    (Equation 27) $$\alpha_{i}=\frac{{\Delta t}_{V_{i}}}{\gcd\left({\Delta t}_{V_{1}},{\Delta t}_{V_{2}}\right)}$$
    where ${\Delta t}_{V_{i}}$ is the time-increment of trace *V*_*i*_. For identical ${\Delta t}_{V_{i}}$, Equation 26 reduces to that given in refs.^21^^,^^88^
    ***Note:*** Different time increments between *apo*, *holo*, and MHQ dipolar traces were not found to perturb the estimated coefficients. However, consider acquiring all traces with a consistent time increment to prevent artifacts due to under- or over-sampling.
  c. Scaled “*apo*-state” and “*holo*-state” dipolar traces are used as components for linear combination fitting of the “mix-state” dipolar trace.^1^^,^^68^
  d. Fit a first-order polynomial to the residual and note the intercept and slope.
    ***Note:*** A slope and an intercept of the fit of approx. zero is a good indicator that the residual is dominated by thermal noise.
  e. Tabulate the coefficients of the *apo*-state and the *holo-*state for subsequent kinetic analysis.
    ***Note:*** Systematic benchmarking of the performance of the deconvolution method will be published elsewhere. Preliminary results indicate that (i) for well-separated and defined distance distributions (i.e., relatively narrow peaks that do not overlap), an SNR of ≥20 is sufficient to determine the coefficients to ±5% accuracy; (ii) for broad distance distributions (i.e., broad peaks that may partially overlap), an SNR of ≥100 is necessary to determine the coefficients to approx. ±5% accuracy.

**Table 8:** PulseSPEL parameters for the inversion-recovery setup experiments

Parameter	Value
p0	12 ns
p1	24 ns
p2	24 ns
d0	360 ns
d1	200 ns
d2	400 ns
d30	8 ns
h	1
n	1
SRT	2000 ∗ srtu (srtu = 1.02 μs)

**Table 9:** PulseSPEL parameters for the inversion-recovery experiment

Parameter	Value
p0	12 ns
p1	24 ns
p2	Determined at step 17c, sub-step x
d0	Determined at step 17c, sub-step vii
d1	200 ns
d2	400 ns
d30	250 μs
h	1
n	3
SRT	200740 ∗ srtu (srtu = 1.02 μs)

**Table 10:** PulseSPEL parameters for the two-pulse ESEEM experiment

Parameter	Value
p0	12 ns
p1	24 ns
d0	432 ns
d1	200 ns
d30	8 ns
h	10
n	3
SRT	2000 ∗ srtu (srtu = 1.02 μs)

## Expected outcomes

The E289R1/I340R1 CNBD-MBP fusion-protein construct expresses at yields of ∼10 mg L^-1^ of bacterial culture. We chose to use an MBP fusion protein to improve expression yield, solubility, and correct protein folding. The protein is purified by a combination of amylose affinity chromatography and subsequent size-exclusion chromatography (SEC). Eluting the protein from the affinity column via denaturation, rather than excess amylose, increases accessibility of cysteine residues for MTSSL spin labeling and improves the labeling efficiency (successful labelling should yield efficiencies >90%). The protein is refolded before loading onto the SEC column. SEC yields a monodisperse CNBD-MBP peak at ∼ 84 mL (Figure 1A). The protein purity can be confirmed by SDS-PAGE, yielding a band at 57 kDa, if successful (Figure 1B). If label conjugation is successful, the CW-EPR spectrum will indicate nitroxide immobilization (Figure 1C).

Optimize the mixer-arm wait-time using methylene blue, with the dilution measured via UV-visible spectrophotometry (Figure 2). Any soluble dye with a known extinction coefficient can be used as a substitute. If no UV-visible spectrophotometer is available, MTSSL may also be used and the spin concentration quantified by CW-EPR. While such optimization is somewhat tedious, it is important whenever the sample concentration is limited (i.e., if the MHQ sample acquisition is performed at low concentrations and small volumes). The dilution behavior as a function of the mixer-arm wait-time is well approximated by a parabolic function with a broad minimum (i.e., for a total sample volume of 200 μL, mixer-arm wait-times of 3.5–6.5 s yield dilution factors of 2-3).

Indeed, the minimum dilution should be observed at a mixer-arm wait-time of 4.5 s, consistent with theoretical predictions. If discrepancies arise between the optimal mixer-arm wait-time and the theoretical prediction, review calculations of both the mixer dead time (*t*_*d*_) and the transit time from the sample-loops to the mixer ($t_{s\to m}$). Importantly, the dilution through the MHQ micromixer is not perfectly two-fold (in the case of 1:1 mixing), but rather three-fold (likely arising from the MHQ dead volume being of the same order of magnitude as the total sample volume). To address the sample dilution that occurs during the transit from the sample loops to the micromixer, a dilution model taking diffusion into account would be ideal, but lies beyond the scope of the current study.

Dilution phenomena are well-established in MHQ sample acquisition - with dilution factors typically ranging between 6-fold and 10-fold from the initial concentration to the concentration inside the EPR tube.^14^^,^^87^ Despite this, few strategies exist to mitigate these effects. Aggregate dilution during MHQ sample acquisition can limit the sensitivity of measurements and impose a lower bound on initial protein and ligand concentrations. To address this, it is useful to factorize the aggregate dilution into contributions from three steps: (i) MHQ mixing, (ii) cold-plate sample deposition, and (iii) sample-packing. By considering the aggregate dilution as a product of these three terms, targeted mitigation may be possible.

Methylene blue dye serves as a reporter for dilution at each step,^13^ enabling three distinct sets of shooting experiments: shooting (i) directly into a 15 mL Falcon tube from the mixer, (ii) onto a pre-cooled cold-plate before collection and subsequent thawing (cold-shooting without packing), and (iii) onto a pre-cooled cold-plate before packing into an EPR tube and thawing (cold-shooting with packing). The corresponding UV-visible spectra averaged from replicate measurements (i.e., for warm-shooting and cold-shooting without packing, *n*=3; for cold-shooting with packing, *n*=2) are shown in Figures 2A, 2C, and 2D, respectively.

As can be seen from Figure 2A, dilution from MHQ mixing, expected to be two-fold from nominal 1:1 mixing, is close to three-fold when using the optimal mixer-arm wait-time. Sample deposition onto the cold-plate adds approximately a three-fold dilution (Figure 2C), while sample packing contributes approximately 1.1-fold dilution (Figure 2D). Such differences in the contribution likely result from water vapor condensation and water-ice formation on the cold-plate before scraping off the sample. The MHQ lid must be removed to fill the cold-plate with N_2(l)_, allowing warm ambient air to displace the nitrogen atmosphere upon exposure to ambient air. This explains the minor dilution during sample packing due to the sample already being saturated with moisture from the air, since the surface area of the cold-plate exceeds that of the funnel or the EPR tube.

This breakdown of aggregate dilution highlights moisture condensation on the cold-plate as the primary dilution source. Potential solutions include adding desiccants under the MHQ lid or in the nitrogen gas used to restore atmospheric pressure before lid removal. Another option is designing a lid that allows delivery of N_2(l)_ to the cold-plate after vacuum release without lid removal. To reduce the three-fold MHQ mixing dilution, decreasing the dead volume of the MHQ (approximately 95 μL per channel, mainly from the PTFE sample tubing) by shortening the length of the tubing is another option. However, theoretically, this can only reduce dilution by about 1.5-times assuming ideal 1:1 mixing.

**Figure 3:**
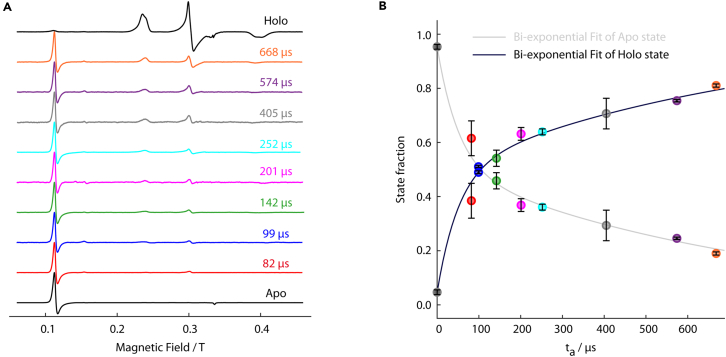
MHQ aging time calibration (A) CW-EPR spectra of the metmyoglobin-azide reaction at different aging times (*t*_*a*_). All spectra were recorded at X-band (9.4 GHz) at 20 K. (B) The state fractions (scatter plots) of the *apo*- and *holo*-state metmyoglobin (i.e., corresponding to signals of *high*- and *low*-spin Fe(III) at ∼0.1 and ∼0.3 T, respectively) as a function of *t*_*a*_. The color scheme is consistent with panel (A). Error bars indicate the 2σ confidence intervals (*n*=3 replicates). The gray and black lines are biexponential fits to the state fractions using Equation 18. Here, the first rate-constant ($k_{1}^{\prime }$) is 19,669 ± 5,406 s^−1^ (pre-exponential coefficient: A = 0.44 ± 0.06) and the second rate-constant ($k_{1}^{\prime }$) is 1,358 ± 189 s^−1^ (pre-exponential coefficient: B = 0.51 ± 0.05). R^2^ of the fit is 0.99.

For calibrating MHQ aging-times, the metmyoglobin-azide reaction is used as a “molecular timer”,^2^ a standard approach for calibration of freeze-quench devices in the millisecond and microsecond regime.^17^^,^^20^ Perform X-band CW*-*EPR measurements at 20 K. In the *apo*-state, the EPR spectrum is axial with features at 0.1 T (*g*_*xx*_=*g*_*yy*_= 5.8) and 0.35 T (*g*_*zz*_= 2.0), characteristic of *high*-spin ($S=\frac{5}{2}$) Fe(III) in the heme moiety. In the *holo*-state, the spectrum is rhombic with features at 0.25 T (*g*_*xx*_= 2.8), 0.3 T (*g*_*yy*_= 2.2), and ∼0.4 T (*g*_*zz*_= 1.8), characteristic of *low*-spin ($S=\frac{1}{2}$) Fe(III) in the heme moiety (Figure 3A). Of note, for MHQ samples, a low-intensity peak appears at ∼0.15 T (*g* = 4.3), indicative of free Fe(III). This peak will intensify if the sample was partially thawed during acquisition, suggesting that a fraction of metmyoglobin can denature and release Fe(III).

As the reaction advances, signals for *high*-spin ($S=\frac{5}{2}$) Fe(III) diminish, while those for *low*-spin ($S=\frac{1}{2}$) Fe(III) increase. For quantification, we recommend focusing on the most intense line of the *low*-spin Fe(III) spectrum - the *g*_*yy*_= 2.2 component. The normalization factor used (15.49) depends on the spectrometer and measurement parameters, and should be calculated case-by-case using Equation 17. The fractions of *apo*- and *holo*-state are expected to follow mono-exponential kinetics. However, experimental factors – such as slight temperature drifts along the MHQ jet, minor pH and concentration variations between samples, and indistinguishable binding modes between N_3_^-^ vs. HN_3_ – result in a bi-exponential behavior (Figure 3B). In a successful measurement series, the determined rate constants should align well with literature values, confirming proper calibration of the MHQ device. As an additional control during initial calibration, we recommend conducting two measurements at different pH values. Self-consistency across these conditions reinforces the accuracy of the device, especially since the kinetics of the metmyoglobin-azide reaction is highly sensitive to pH (e.g., pH 5^1,2^ and pH 7.8^20^).

**Figure 4:**
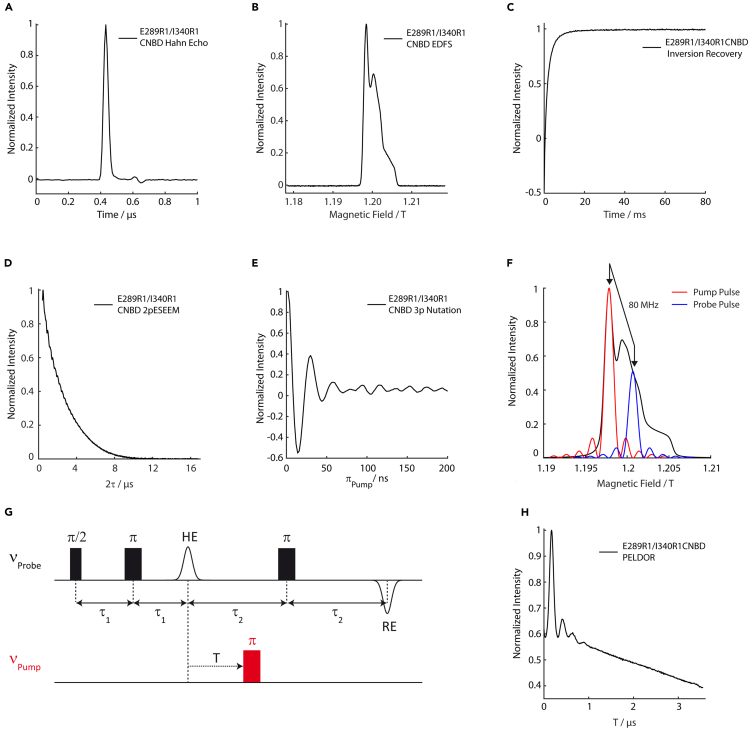
PELDOR experimental setup and pulse sequence (A) Hahn echo transient; pulse sequence *π*/2-*τ*-*π*-*τ*-*echo*. (B) Echo-detected field-swept EPR spectrum. (C) Inversion recovery trace; pulse sequence *π_inv_*-*T*-*π*/2-*τ*-*π*-*τ*-*echo*. (D) Two-pulse ESEEM trace; pulse sequence *π*/2-*τ*-*π*-*τ*-*echo*. (E) Transient nutation trace; pulse sequence *π*_*nut*_-*t*_1_-*π*/2-*t*_2_-*π*-*t*_2_-*echo*. (F) Echo-detected field-swept EPR spectrum at Q-band of a nitroxide spin center (black) and the excitation profiles of the PELDOR pump pulse (red, 24 ns) and probe pulse (blue, 24 ns). The frequency offset between the pump pulse and the probe pulse is 80 MHz. (G) The four-pulse PELDOR experiment pulse sequence. The experiment is a two-frequency (i.e., pump-probe) approach, where the magnetization of probe-spin A is perturbed through dipolar coupling to pumped spin B. The temporal position of the pump pulse is incremented, yielding a refocused echo (RE) modulated with the dipolar-coupling frequency as a function of time *T*. (H) PELDOR dipolar trace (raw data); pulse sequence: *π*/2_*probe*_-*τ*_1_-*π*_*probe*_-*T*-*π*_*pump*_-(*τ*_2_-*T*)-*π*_*probe*_-*τ*_2_-*echo*. All measurements are illustrated using the example of CNBD construct C263S/E289R1/C331S/I340R1 in the *holo*-state at 50 K at Q-band.

When preparing a PELDOR experiment, the first step is typically to detect and optimize the Hahn echo transient (Figure 4A). At this stage, carefully adjust the video gain, high-power attenuation, microwave phase, and shot-repetition time (SRT) to optimize the signal, preventing both signal clipping (avoid saturating the SpecJet oscilloscope) and saturation of the spin system (ensure SRT remains above the longitudinal electron-spin relaxation time, *T*_1_). Validate that the chosen SRT is optimal by performing an inversion-recovery (InvRec) experiment (Figure 4C) to measure *T*_1_, confirming that the SRT is at least 5 × *T*_1_.

Next, identify the maximum of the nitroxide EPR spectrum by measuring the integrated Hahn echo intensity as a function of the external magnetic field, resulting in the echo-detected field-swept (EDFS) EPR spectrum (Figure 4B). At Q-band in frozen solution at 50 K, the EDFS EPR spectrum of a nitroxide is typically dominated by g-tensor anisotropy, displaying a low-field maximum (associated with a mixture of *g*_*xx*_ and *g*_*yy*_), and a high-field shoulder (associated with *g*_*zz*_). To determine the transverse electron-spin relaxation time (phase-memory time, *T*_*M*_) and to select an appropriate dipolar evolution time for the PELDOR experiment (which defines the time interval in which dipolar-coupled electron spins accumulate a phase-offset, corresponding to the length of the dipolar trace), a two-pulse electron spin echo envelope modulation (two-pulse ESEEM, Figure 4D) experiment is performed. Subsequently, determine the optimal pump-pulse length, using a transient nutation experiment (Figure 4E) to find the pulse length that achieves maximal inversion of magnetization (the global minimum of the nutation trace).

PELDOR is a double-resonance pump-probe technique that uses two microwave frequencies. To facilitate this, a frequency offset – typically 80-100 MHz at Q-band – is applied between the “probe” (A-spins) and “pump” (B-spins) spin packets (Figure 4F). The pump pulse is positioned at the maximum of the nitroxide spectrum to ensure efficient inversion, thereby optimizing modulation depth and sensitivity. The PELDOR pulse sequence at the probe frequency, ν_probe_, begins with a preparation subsequence that produces a Hahn echo, marking the zero-time in the dipolar evolution, i.e., the maximum of the dipolar trace (Figure 4G). At the pump frequency, ν_pump_, an inversion pulse is time-incremented to invert the magnetization of the B-spin packet. B-spins dipolar-coupled to the A-spin packet then alter the A-spin magnetization. Applying a π-pulse at ν_probe_ refocuses the magnetization, producing a refocused echo that oscillates in time with the dipolar coupling frequency. Measuring the refocused echo as a function of the dipolar evolution time generates a dipolar trace (Figure 4H) that encodes the intramolecular dipolar coupling as oscillations. This pattern is further convoluted with intermolecular dipolar coupling, forming the background decay of the trace.

**Figure 5:**
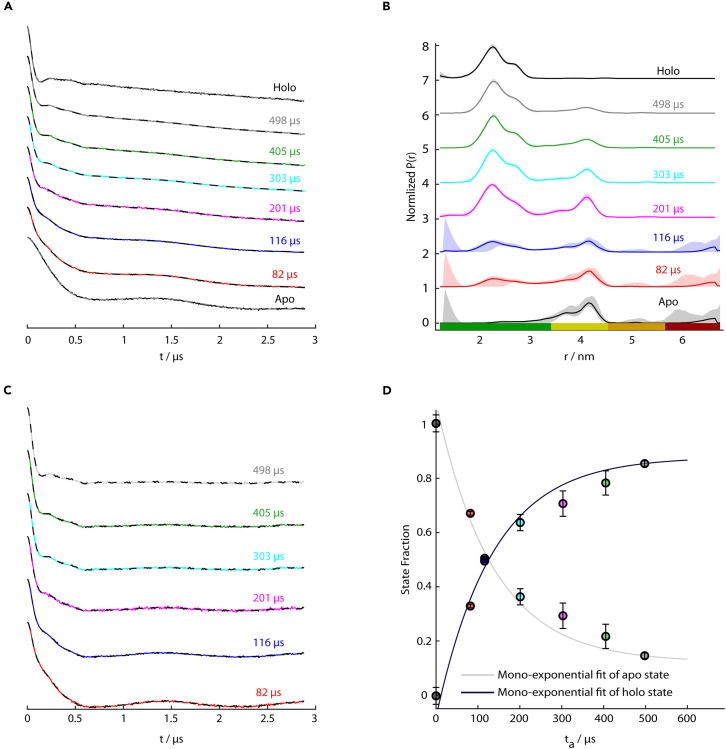
PELDOR measurements and data analysis (A) PELDOR raw dipolar traces obtained at different MHQ aging times (*t*_*a*_). The dashed lines correspond to a fit of the intra- and intermolecular contributions of the dipolar coupling to the experimental traces. All measurements were performed at Q-band frequency (34.0 GHz) and 50 K. (B) Distance probability-density distributions obtained from the PELDOR traces at different MHQ aging times. Data analysis was performed using the standalone ComparativeDeerAnalyzer 2.0. (C) Linear-combination fitting of MHQ/PELDOR dipolar traces as a weighted sum of *apo*- and *holo*-state dipolar traces. Colored traces indicate experimental data, dashed lines indicate the *in-silico* mixtures. (D) Fractions of the *apo*- and *holo*-state as a function of the aging time (scatter). Error bars indicate the 2σ confidence intervals (*n*=3 replicates). The gray line is a mono-exponential fit ($y=y_{0}+A\cdot e^{-{kt}_{a}}$) to the *apo*-state fraction, where the black line is the gray line subtracted from unity. Here, y_0_ = 0.12 ± 0.04, A = 0.96 ± 0.08, k = 7,398 ± 1,179 s^−1^. The color scheme is consistent with (C).

After calibrating the MHQ aging-times, the CNBD was mixed with cAMP in the MHQ device, followed by PELDOR measurements on each sample. Figure 5A shows the raw dipolar traces collected from CNBD samples prepared at various aging-times, with the corresponding distance distributions shown in Figure 5B. We analyzed the dipolar traces using the ComparativeDeerAnalyzer 2.0 to reduce confirmation bias. As expected, the *apo*-state population (with a probability density peak near ∼4 nm) decreases over time, while the *holo*-state population (with a probability density peak near ∼2 nm) simultaneously increases with the aging-time. Because the *apo* and *holo* populations are well-separated by distance, state fractions can be calculated directly by integrating the respective peaks. To mitigate the impact of Tikhonov regularization on these fractions, the MHQ/PELDOR dipolar traces were fitted as linear combinations of *apo*- and *holo*-state traces (Figure 5C).

The packing procedure for MHQ/PELDOR sample powder can sometimes lead to a non-homogeneous sample distribution in the EPR tube, potentially causing deviations in the intermolecular background of the PELDOR signal from a theoretical three-dimensional homogeneous (mono-exponential) decay.^22^ In some instances, importing raw dipolar traces into the deconvolver GUI produced negative (non-physical) weights in the linear combination fitting; this issue can be resolved by using background-corrected PELDOR traces obtained from DEERNet. The quality of the linear-combination fits is excellent (R^2^ > 0.99), reinforcing the conclusion that no intermediate forms were captured during the transition from *apo*- to *holo*-state.

Finally, a mono-exponential function was fitted to the *apo*- and *holo*-state fractions obtained from the linear combination analysis (Figure 5D). The resulting rate constant can be interpreted as the average dwell-time that the ligand-bound *apo*-state occupies before transitioning to the *holo*-state, reflecting the timescale of the cAMP-induced conformational change rather than the kinetics of cAMP binding or individual conformational transitions. At shorter aging times, fewer cAMP-bound *apo*-state CNBD molecules have transitioned to the *holo*-state, resulting in a lower *holo*-state population. The measured average dwell time is 135 ± 22 μs, which aligns well with the time resolution of the MHQ device. However, to capture subtle conformational events such as coordinated motions of specific secondary-structure elements, further improvements in temporal resolution, reaching into the low nanosecond range, will be required.

## Limitations

The primary limitation of microsecond freeze-hyperquenching lies in its high material cost. First, resolving conformational kinetics requires system equilibration to steady-state conditions before cryofixation to uphold assumptions of pseudo-first order kinetics. Second, dilution at each stage of MHQ sample acquisition requires initially high protein (spin) concentrations to obtain high-quality PELDOR data. For the shortest aging-time point (82 μs in this study), a substantial excess of ligand compared to protein is necessary, making ligand availability and cost important considerations; for example, a 66-fold molar excess of cAMP to CNBD was required for rapid equilibration prior to cryofixation at 82 μs.^1^ Furthermore, cryofixation onto the cold-plate (approximately 40 μs) also limits time-resolution to the mid-μs range. Advanced approaches, such as nanosecond freeze-hyperquenching (NHQ), bypass the cold-plate to achieve higher time resolution.^89^ Completing a full time series (excluding technical triplicates) typically requires 5-6 samples, as well as *apo*- and *holo*-state controls, leading to ∼30 total measurements when replicates are included.

Another limitation of MHQ sample acquisition is the high pressure within the mixer and the orifice, which can perturb protein conformational equilibria, as documented in pressure-dependent EPR^90^^,^^91^ and NMR^92^ studies. Elevated pressure risks shifting protein structures from their global Gibbs free energy minimum into metastable or non-native states, potentially causing aggregation or precipitation – a problem exacerbated by protein over-concentration, which may also compromise solubility. The PELDOR technique itself presents challenges for MHQ samples, as it is highly sensitive to the local concentration of electron spins. Its pump-probe design limits the achievable modulation depth to ∼30% at Q-band, which in turn restricts both the detectable spin concentration and the resulting signal-to-noise ratio (SNR).

Importantly, introducing protons into the MHQ sample via dilution can markedly shorten the electron-spin phase-memory time (*T*_M_), further lowering the SNR and limiting both the maximum measurable distance and the reliable interpretation of distance distributions. Although rapid freeze-quenching (RFQ) below one millisecond can prevent formation of crystalline ice without cryoprotectant,^3^ accurate modelling of the intermolecular background function (to extract intramolecular interspin distances) depends on a homogeneous glass – a condition not always met. Using a deuterated cryoprotectant can mitigate elevated proton concentration and non-homogeneous sample distribution; however, increased sample viscosity may impede thorough mixing.

To minimize dilution, it is crucial to reduce condensation of moisture during each stage of MHQ sample acquisition, particularly when cooling the cold-plate. Following sample deposition onto the cold-plate, the vacuum lid is typically removed before submerging the cold-plate in N_2(l)_, a process that allows rapid moisture condensation from ambient-temperature air. One solution is filling the cold-plate with N_2(l)_ while the vacuum lid remains mounted, maintaining a cold nitrogen atmosphere and preventing exposure to ambient temperature. A newly designed vacuum lid with a sufficiently large valve would enable rapid filling of the cold-plate with N_2(l)_. Additionally, sample packing in an environment with controlled, very low humidity can further reduce sample dilution at this stage.

To address low SNR and allow for lower concentrations of protein and ligand, consider alternative PDS methods. Single-resonance techniques such as relaxation-induced dipolar modulation enhancement (RIDME)^93^^,^^94^^,^^95^ or double quantum coherence (DQC)^96^ may offer higher sensitivity than PELDOR. However, RIDME is affected by high proton concentration in MHQ samples (owing to dilution from air moisture), which can increase electron-nuclear spectral diffusion during the mixing block (*T*_*mix*_).^93^^,^^94^^,^^97^ DQC requires uniform excitation across the full EPR spectrum, which is challenging for nitroxides when using conventional rectangular pulses. Nevertheless, DQC achieved nanomolar concentration sensitivity when combined with species exhibiting an exceptionally narrow spectral width such as trityls^61^^,^^98^ – especially with a loop-gap resonator and 300 W TWT amplifier.^99^ Other promising strategies include variable-time acquisition schemes,^100^^,^^101^ non-uniform sampling routines,^102^ and the use of cryogenically-cooled preamplifiers.^103^^,^^104^

Furthermore, shorter aging times may be reached by (i) decreasing the jet diameter using a narrower orifice, thereby reducing the cryofixation time on the cold-plate and (ii) increasing the thermal conductivity of the cold-plate using materials like strips of diamond or carbon nanotube networks.^105^ Concomitantly, the time-of-flight in the jet could be reduced by increasing the flow rate (and therefore the jet velocity) by using system pressures greater than 2000 psi. This approach would require replacing platinum with a stronger orifice material. Moreover, shortening the distance between orifice and cold-plate further decreases the jet length; in this scenario, a rounded, concave nozzle is needed to prevent copper abrasion from the cold-plate surface.

## Troubleshooting

### Problem 1

Over- or underpressure in the HPLC pumps due to blockage or leakage of tubing. See step 21, step 22, step 37a, and step 41.

### Potential solution

Overpressure typically results from blockage within the tubing caused by either particulate matter or air bubbles in the buffer solutions. To prevent this, all buffer solutions should be double-filtered and degassed using a 0.45 μm filter and re-filtered on the day of use. Freezing of tubing can occur when cooling the cold-plate with N_2(l)_, especially since the tubing leading to the micromixer runs partially above the cold-plate along the mixer arm. This may cause a sudden pressure spike in the HPLC pumps. To prevent this, insulate the tubing and minimize N_2(l)_ spillage outside the cold-plate during cooling. If freezing occurs, thaw the tubing with a hairdryer and restart the cooling procedure. If a split jet appears at the nozzle following a pressure spike, the platinum orifice may have deformed (common at pressures > 2000 psi). In this case, dismount and replace the orifice. Underpressure is most often due to leakage. During sample injection from the loops into the micromixer, a sudden pressure change may cause the tubing to detach from the connectors and cause sample loss. Always use HPLC- or UHPLC-grade connectors, tighten and inspect them regularly, and be aware that tubing can loosen over time, increasing dead volume between the injector and the micromixer.

### Problem 2

Sample ejection from the EPR tube after packing. See step 12, step 45, step 52, and step 53.

### Potential solution

Sample ejection can arise from low sample packing density, which creates voids within the EPR tube that may trap nitrogen gas (particularly during long-term storage in a storage Dewar) or from exposure to ambient temperature when inserting the EPR tube into the cryostat of the spectrometer. This issue can be addressed by annealing the sample in cold isopentane (<−130°C) before mounting the tube in the spectrometer, which removes trapped nitrogen from the voids. A 5-10 min annealing step, combined with repacking the sample to compress and eliminate such voids typically suffices to prevent sample ejection. For sample packing, use a rod cryogenically cooled in a separate isopentane reservoir (<−130°C), rather than one cooled in N_2(l)_. Always perform this annealing and repacking procedure whenever an MHQ sample is taken from long-term storage.

### Problem 3

Poor signal-to-noise ratio in the PELDOR measurements. See step 19, and step 25f.

### Potential solution

Poor signal-to-noise ratios can arise from several sources: (i) poor labeling efficiency, (ii) excessive sample dilution resulting in reduced electron spin concentration, and (iii) high proton density in the sample matrix, which accelerates spin-echo decay. Aim for near-quantitative labeling to ensure reliable analysis of conformational kinetics. If a consistent labeling efficiency >85% is unattainable, consider redesigning the protein construct to provide more accessible labeling sites. To counteract sample dilution, calibrate wait-times with methylene blue and optimize mixer-arm timing, which markedly increases the amount of sample delivered to the cold-plate. Furthermore, during funnel cool-down, place a dry towel into the inner funnel to prevent air moisture from condensing in the EPR tube. Reduce the proton density of the sample matrix by using deuterated cryoprotectant and consider using small volumes (approx. 20 mL per channel) of per-deuterated buffers during MHQ sample acquisition. Nonetheless, in this protocol moisture condensation onto the cold-plate is the primary cause of sample dilution; therefore, attempt first to minimize the condensation on the cold-plate before working with per-deuterated buffers.

### Problem 4

The vacuum lid fails to seal; no vacuum is achieved. See step 26h, step 41a-d, step 52.

### Potential solution

To reduce air moisture condensing on the cold-plate, minimize the time between removing N_2(l)_ from the cold-plate (after cooling) and sealing the vacuum lid. Difficulty in achieving a tight seal can arise from the lid O-ring coming loose, moisture on the MHQ surface, or improper lid orientation and placement. To secure the O-ring, affix tape at several points around the lid to hold it inside the groove, while positioning the lid over the cold-plate. Avoid using vacuum grease, as it complicates lid handling (i.e., holding, carrying, orienting) and does not resolve the problem. If the contact surface of the MHQ—where the lid will sit over the cold-plate—becomes wet or damp during cold-plate pre-cooling, dry it thoroughly with a fresh towel before commencing sample acquisition. Finally, should the lid become misaligned, simply rotate it in-place to realign. If the vacuum still does not seal and sample is retained in the Hamilton syringes, return these to ice, warm up and clean the cold-plate, and restart the pre-cooling and sealing procedure.

### Problem 5

Static electricity disrupts the jet path. See step 22, step 41.

### Potential solution

High jet velocity (up to 200 m s^-1^)^2^ can lead to localized buildup of charge density if the materials involved are electrically non-conducting, creating strong electrostatic repulsion between the mixer-arm and the cold-plate or waste collector (with voltages reaching up to 10 V). This repulsion causes the jet to deflect onto itself and the nozzle; eventually resulting in a split jet. Sample particles then become harder to pack, tending to stick to the tube walls, and the risk of static shock to the operator increases. To address this, use a metallic waste collector (not a plastic one) and always ensure the mixer-arm is properly grounded to allow static charge dissipation. These measures also ease sample packing.

## Resource availability

### Lead contact

Further information and requests for resources and reagents should be directed to and will be fulfilled by the lead contact, U. Benjamin Kaupp (ubkaupp@uni-bonn.de).

### Technical contact

Technical questions on executing this protocol should be directed to and will be answered by the technical contact, Joshua L. Wort (joshua.wort@manchester.ac.uk).

### Materials availability

This study did not generate new unique reagents.

### Data and code availability

Underpinning data have been deposited at Zenodo and are publicly available as of the date of publication at https://doi.org/10.5281/zenodo.18524338. All original code has been deposited at Zenodo at https://doi.org/10.5281/zenodo.18436098 and is publicly available as of the date of publication. Any additional information required to reanalyze the data reported in this paper is available from the lead contact upon request.

## Acknowledgments

Funding by the 10.13039/501100001659DFG via a Reinhart Koselleck Grant (Projektnummer 420322655) to U.B.K. and the 10.13039/501100008131University of Bonn via TRA-2 (Building Blocks of Matter and Fundamental Interactions) to O.S. is gratefully acknowledged. O.K. gratefully acknowledges funding from the 10.13039/501100001645Boehringer Ingelheim Fonds PhD fellowship. We thank Dr. Tobias Zbik, Dr. Reinhard Seifert, Dr. Sebastian Peuker, and Norbert Brenner for their valuable contributions at the initial stage of this project. We especially thank Dr. Wolfgang Bönigk for designing and providing the primers for the CNBD constructs.

Open Access funding enabled and organized by Projekt DEAL.

## Author contributions

J.L.W., T.H., U.B.K., and O.S. designed the protocol. J.L.W., T.H., A.H., and O.K. constructed the key resources table. A.H., O.K., and H.A. extensively discussed troubleshooting with J.L.W. and T.H. J.L.W. and T.H. performed the wait-time calibration, wrote the deconvolver GUI MATLAB code, and drafted the manuscript. All the authors revised the manuscript for important intellectual content and approved the manuscript.

## Declaration of interests

T.H. was an employee of Bruker BioSpin GmbH & Co. KG at the time of submission.
