# Supplementary material for: Protocol for preparing and characterizing samples for combined microsecond freeze-hyperquenching and electron paramagnetic resonance spectroscopy
Source: STAR Protoc. 2026 Feb 27;7(1):104405. doi: 10.1016/j.xpro.2026.104405 (PMC12964019; doi:10.1016/j.xpro.2026.104405)
Supplement: Document S1. Figures S1–S5 [file mmc1.pdf]

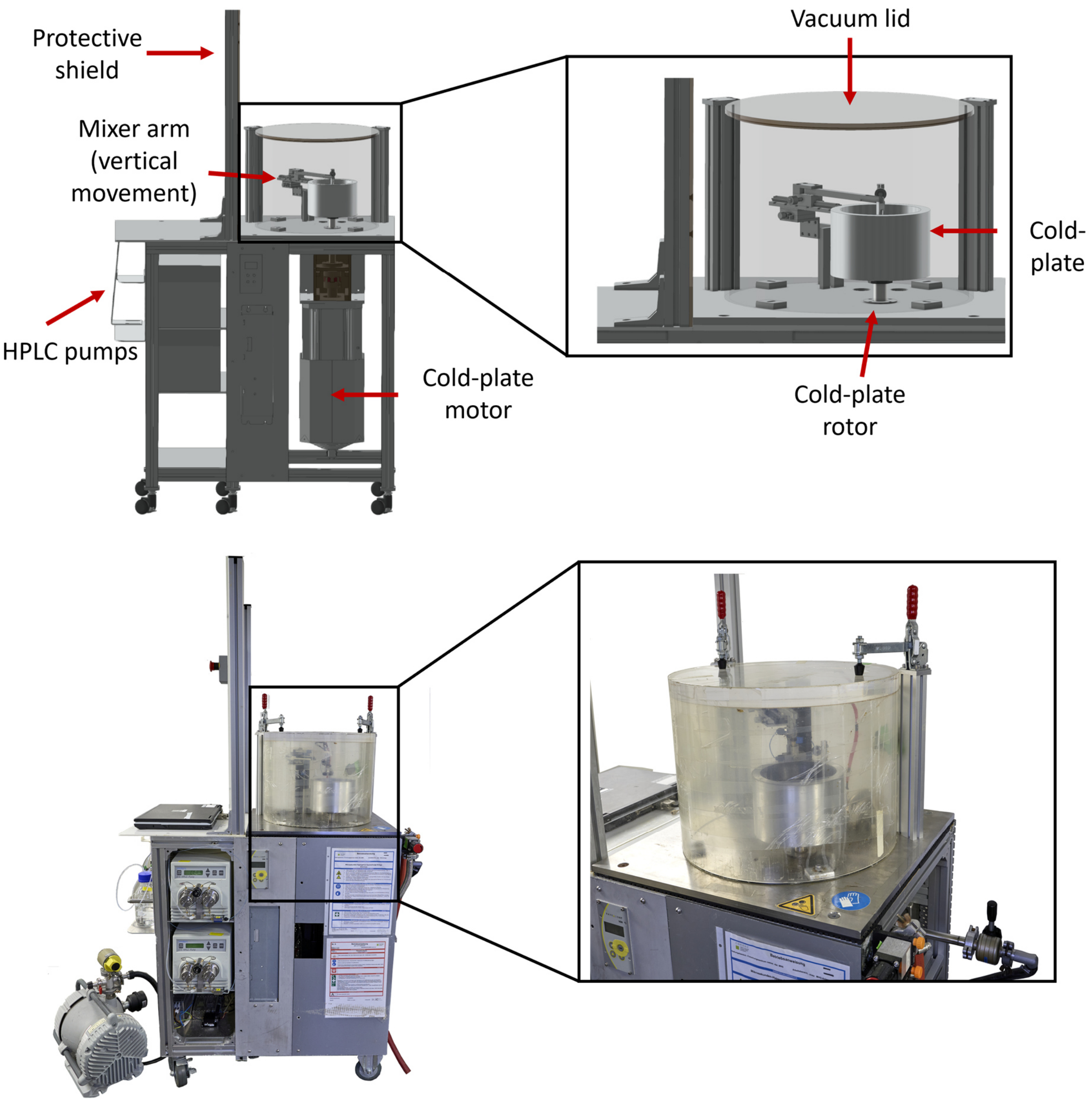

Figure S1: The MHQ device. Related to step 22. (top left) Schematic computer-assisted diagram of the MHQ device. Components of interest are labelled. (top right; inset) Schematic computer-assisted diagram of the MHQ cold-plate, mixer arm, lid, and cold-plate rotor. Components of interest are labelled. (bottom left) The corresponding photograph of the MHQ device. (bottom right; inset) The corresponding photograph (close-up view) of the MHQ cold-plate, mixer arm, and micromixer.

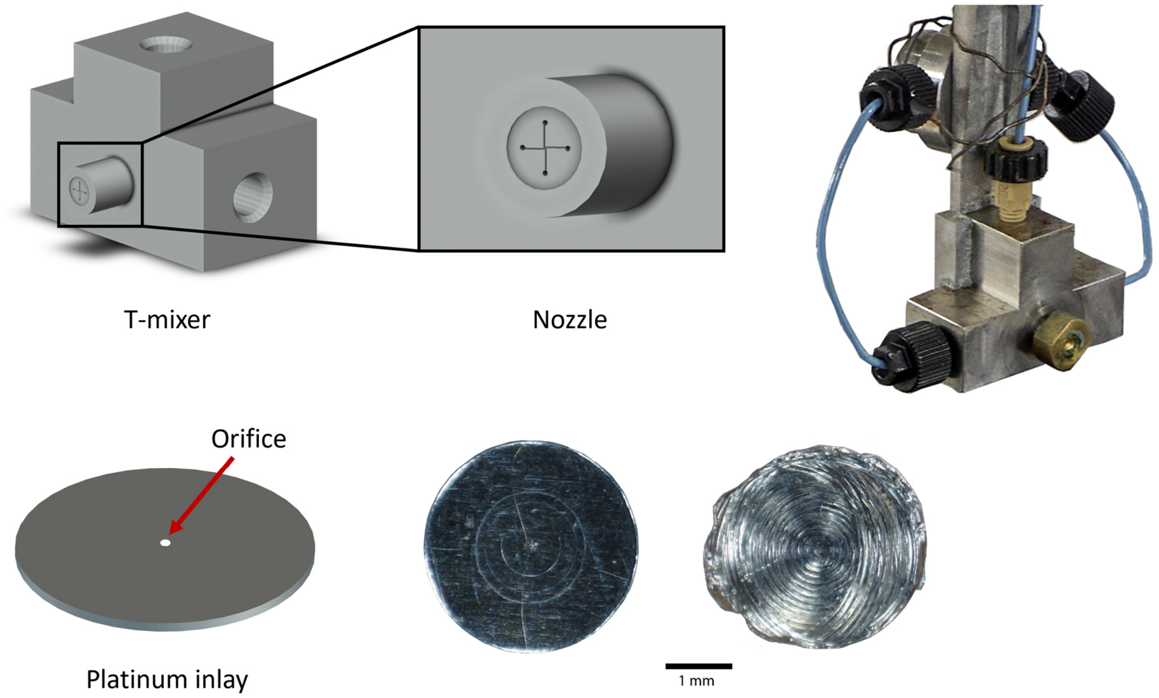

Figure S2: The MHQ micromixer. Related to step 22. (top left) Schematic computer-assisted diagram of the T-type MHQ mixer, and (top left; inset) Schematic computer-assisted diagram of the nozzle in close-up, as indicated by the black box. (top right) The corresponding photograph of the T-type MHQ mixer. (bottom left) Schematic computer-assisted diagram of the platinum inlay with the orifice (diameter: 20 µm) in the center. (bottom right) Micrographs of an intact (left) and damaged (right) platinum inlay, before and after over-pressure. The scale bar corresponds to 1 mm.

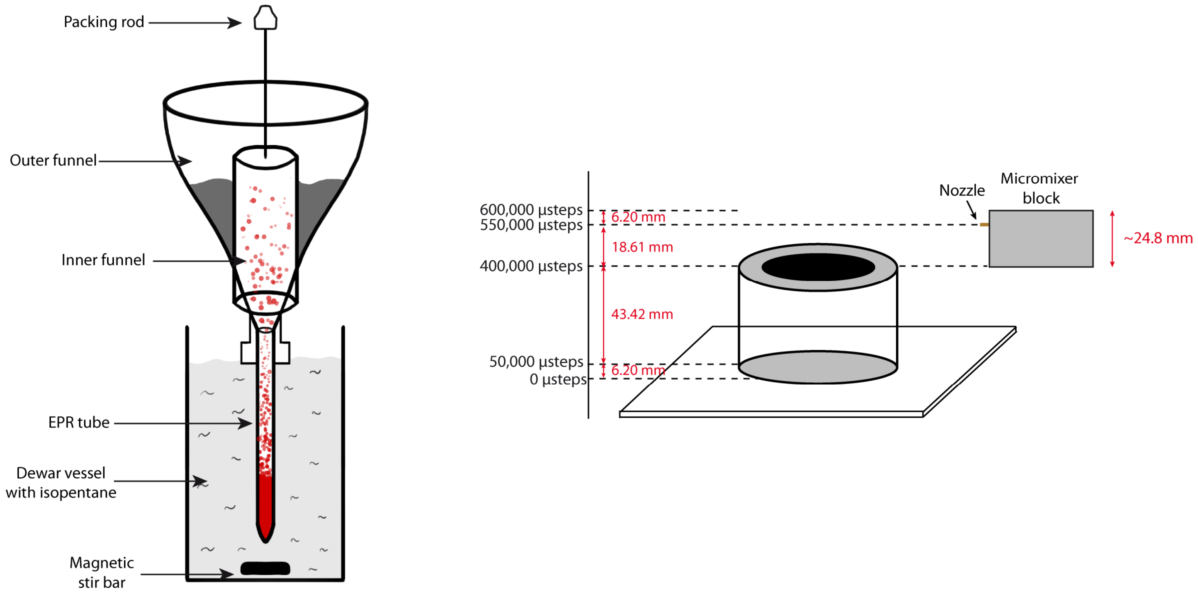

Figure S3: MHQ sample-packing funnel apparatus, cold-plate, and micromixer. Related to steps 26, 30, 34 and 45. (left) Schematic of the sample-packing funnel apparatus. The outer funnel is submerged in $N_{2(l)}$, the inner funnel contains the MHQ sample to be packed. During packing, water ice can accumulate inside the inner and outer funnel and, therefore, contribute to sample dilution. (right) Explanation of the µstep-scale for cold-plate and micromixer. The number of µsteps refers to the position of the nozzle. At 600,000 µsteps, the micromixer block is outside the cold-plate (so-called "home position"). At 400,000 µsteps, the nozzle aligns with the top of the cold-plate, the bottom of the micromixer block is already inside the cold-plate. 50,000 µsteps is the minimal safe position the nozzle can be lowered to without the mixer block touching the cold-plate.

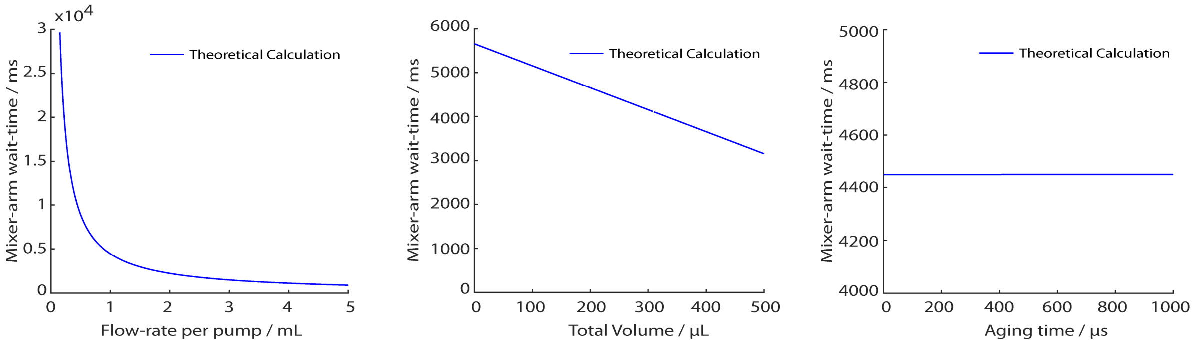

Figure S4: Optimal mixer-arm wait-time as a function of different parameters. Related to step 30. (left) Optimal mixer-arm wait-time simulated for different total flow rates, assuming a fixed sample volume of 200 µL. (center) Optimal mixer-arm wait-time simulated for different total sample volumes, assuming a fixed total flow rate of 2 mL min^{-1}. (right) Optimal mixer-arm wait-time simulated for different aging times, assuming a fixed total flow rate of 2 mL min^{-1} and a fixed sample volume of 200 µL.

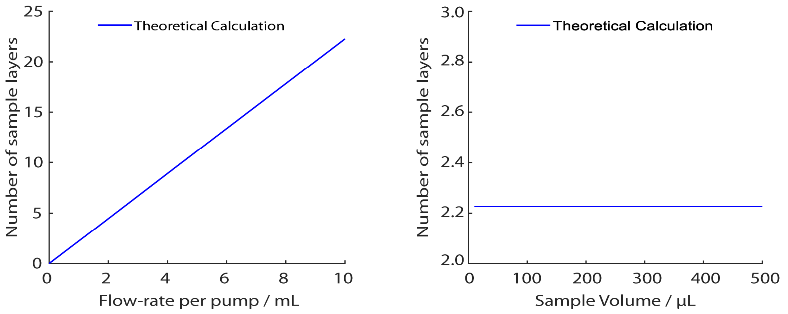

Figure S5: Number of sample layers and layer thickness as a function of different parameters. Related to step 41. (left) Number of sample layers simulated for different total flow rates, assuming a fixed sample volume of 200 µL. (center) Number of sample layers simulated for different total sample volumes, assuming a fixed total flow rate of 2 mL min^{-1}. (right) Sample layer thickness simulated for different total sample volumes assuming a fixed cold-plate radius of 6.5×10^{-2} m.
